# Supplementary material for: Evolution of the crustal phosphorus reservoir
Source: Sci Adv. 2023 May 5;9(18):eade6923. doi: 10.1126/sciadv.ade6923 (PMC10162663; doi:10.1126/sciadv.ade6923)
Supplement: 20230505-1 [file sciadv.ade6923.v1.pdf]

CORRECTION POSTED 21 JULY 2023

GEOLOGY

Evolution of the crustal phosphorus reservoir

Craig R. Walton[1]*, Jihua Hao[2,3]*, Fang Huang[4], Frances E. Jenner[5], Helen Williams[1], Aubrey L. Zerkle[6], Alex Lipp[7], Robert M. Hazen[8], Shanan E. Peters[9], Oliver Shorttle[1,10]

Copyright © 2023 The Authors, some rights reserved; exclusive licensee American Association for the Advancement of Science. No claim to original U.S. Government Works. Distributed under a Creative Commons Attribution License 4.0 (CC BY).

The release of phosphorus (P) from crustal rocks during weathering plays a key role in determining the size of Earth's biosphere, yet the concentration of P in crustal rocks over time remains controversial. Here, we combine spatial, temporal, and chemical measurements of preserved rocks to reconstruct the lithological and chemical evolution of Earth's continental crust. We identify a threefold increase in average crustal P concentrations across the Neoproterozoic-Phanerozoic boundary (600 to 400 million years), showing that preferential biomass burial on shelves acted to progressively concentrate P within continental crust. Rapid compositional change was made possible by massive removal of ancient P-poor rock and deposition of young P-rich sediment during an episode of enhanced global erosion. Subsequent weathering of newly P-rich crust led to increased riverine P fluxes to the ocean. Our results suggest that global erosion coupled to sedimentary P-enrichment forged a markedly nutrient-rich crust at the dawn of the Phanerozoic.

INTRODUCTION

The size of Earth's biosphere is limited by those bioessential elements that are in scarce supply. Many nutrients must first be liberated from surface-exposed continental rocks to be made available to life, creating a link between the composition and surface processing of Earth's crust and the evolution of the biosphere. This is especially true for phosphorus (P), a candidate element for the ultimate limiting nutrient for life on Earth. However, it remains challenging to quantify the concentration of P within Earth's crust over time: This problem requires both identifying the lithology proportions in the crust and the P concentrations of those lithologies.

Increases in P concentration within continental igneous rocks have been suggested to modify the nutrient supply to early life (*1*–*3*). However, to know how this translated into P fluxes to the biosphere, it is key to constrain the rock types that made up Earth's weatherable crust back through time, i.e., the uppermost layer of crust that interacts, via weathering, with the surface environment (*4*, *5*). Planets initially develop crusts solely composed of igneous rocks, yet the portion of Earth's crust that is exposed to weathering is today mostly made up of sedimentary rock (*6*, *7*). The long-term evolution of Earth's crust from broadly igneous to mainly sedimentary is of both unknown timing and uncertain consequence for global nutrient supply.

The major element chemistry of sediments and igneous rocks do appear to have been strongly correlated and static over much of the past several billion years of Earth history (*4*). However, sediments may become strongly enriched in nutrients compared to the igneous rocks from which they ultimately derive, owing to processes that occur during biological cycling (*8*–*12*). For example, P may be preferentially deposited on continental shelves during biomass burial (*13*). Continental crust may therefore become enriched in P over time if those nutrient-rich marginal sediments are retained rather than subducted. Consequently, constraints are needed on continental lithochemical evolution, changes in the relative abundance of crustal rock types (lithologies), and their chemical compositions.

We use the Macrostrat database to reconstruct the lithochemical evolution of weatherable crust using preserved rocks. Macrostrat describes the age, lithology, area, thickness, and associated geochemical measurements of geological units (*10*) in stratigraphic columns. This approach allows for quantification of subsurface rock volumes, leading to estimates of uppermost crustal geology that are plausibly more accurate than map-only analyses. Measurements in Macrostrat can be weighted by the volume of parental rock units when quantifying crustal composition. This approach eliminates the possibility that outlier measurements may skew mean compositional values (*14*).

Preserved rocks may represent a powerful constraint on crustal evolution. However, the utility of the preserved rock record for tracing crustal evolution hinges on whether different lithologies of the same age have been differentially preserved, e.g., due to differences in inherent durability during physical and chemical weathering (*5*, *15*, *16*) or the preferential formation and subsequent exhumation of sediments in marginal settings (*17*–*20*). Here, we show that the rock record contains long (billion-year) periods of lithological equilibrium and is therefore a reliable record of crustal lithochemical evolution. We use our record of lithochemical evolution to calculate a record of overall crustal P concentration over time. We find that sediment chemical evolution acted to decouple the P concentration of weatherable crust from that predicted by igneous chemical evolution alone. Coeval with this chemical shift, massive removal of ancient crust and deposition of young sediment then conspired to enrich crustal P by threefold across the Neoproterozoic-Phanerozoic transition [600 to 400 million years (Ma)].

[1]Department of Earth Sciences, University of Cambridge, Downing Street, Cambridge CB2 3EQ, UK. [2]Deep Space Exploration Lab/CAS Key Laboratory of Crust-Mantle Materials and Environments, University of Science and Technology of China, 96 Jinzhai Rd., Hefei 230026, China. [3]CAS Center for Excellence in Comparative Planetology, University of Science and Technology of China, 96 Jinzhai Rd., Hefei, 230026, China. [4]CSIRO Mineral Resources, Kensington WA 6151, Australia. [5]School of Environment, Earth and Ecosystem Sciences, The Open University, Walton Hall, Milton Keynes MK7 6AA, UK. [6]Blue Marble Space Institute of Science, Seattle, WA 98154, USA. [7]Department of Earth Sciences and Engineering, Imperial College London, London, UK. [8]Geophysical Laboratory, Carnegie Institution for Science, 5251 Broad Branch Road NW, Washington, DC 20015, USA. [9]Department of Geoscience, University of Wisconsin–Madison, Madison, WI 53706, USA. [10]Institute of Astronomy, University of Cambridge, Madingley Road, Cambridge CB3 OHA, UK.
*Corresponding author. Email: crw59@cam.ac.uk (C.R.W.); hao@ustc.edu.cn (J.H.)

RESULTS

Lithological evolution of weatherable continental crust

We limit our analysis of the lithological make-up of Earth's continental crust to those rock units younger than 3000 Ma, beyond which the increasingly low temporal resolution of the rock record (as sampled by Macrostrat) is insufficient to obtain robust samples using our approach. Macrostrat continental data coverage is best in North America, with some limited representation in South America and New Zealand (*7*). However, previously published trends in rock area over time in Macrostrat compare favorably to low-resolution data available on a global scale (*11*).

We obtain estimates of preserved rock volume for siliciclastic (non-carbonate, silicate rich) and carbonate sedimentary, metasedimentary, and igneous rocks (Fig. 1, A to D). The fraction of cumulative rock volume for each rock type is used to reconstruct the lithological composition of weatherable crust. The results of this calculation are presented in Fig. 1E.

Preserved igneous, siliciclastic, and carbonate rock volumes all display similar trends. Volumes increase linearly over much of Earth history, except for sharply across the Neoproterozoic-Phanerozoic boundary (600 to 400 Ma) (Fig. 1, A to D) (*7*). Metasediments are an exception, declining toward the present day—an observation consistent with the necessary time taken to bury and metamorphose sediments (Fig. 1D). While it is difficult to partition metasediments systematically due to uncertainties in the formation conditions of their precursor materials, it is worth noting that the early Phanerozoic peak in metasediment volume is partially driven by abundant quartzites, which are of clear siliciclastic origin.

Considering the overall lithological make-up of preserved crust and building on earlier studies (*6*, *12*), we find that the fraction of cumulative rock volume represented by siliciclastic sedimentary and metasedimentary rocks begin to substantially increase after 3000 Ma, undergoing a sharp up-tick until 2400 Ma (Fig. 1E). The fraction of preserved rock as carbonate first begins to increase at around 2800 Ma, displaying no inflection across the Archean-Proterozoic boundary (Fig. 1E). Cumulative rock volume proportions are then essentially invariant over the middle 1.5 billion years of Earth history (Fig. 1E). Carbonate, siliciclastic, and metasedimentary rocks then increase steeply in abundance relative to igneous rocks across the Neoproterozoic-Phanerozoic boundary (600 to 400 Ma; Fig. 1E).

The Archean to Paleoproterozoic (3000 to 2400 Ma) increase in sedimentary proportion is understandable if sediment storage and/or erosion rates increased in this interval, consistent with a late Archean onset of modern-style plate tectonics (*21*, *22*). Our results suggest that igneous crust was the most abundant component of Archean weatherable continental crust, with sedimentary rocks coming to dominate only after 2500 Ma.

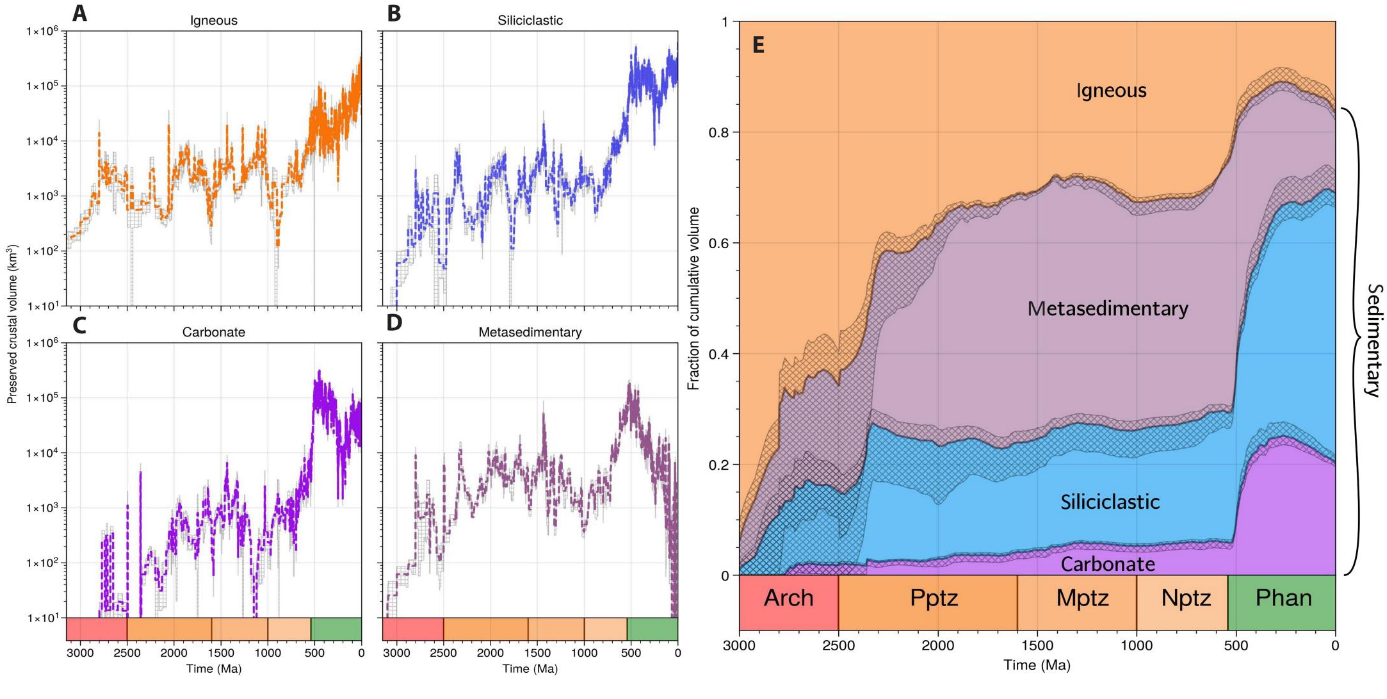

Fig. 1. Trends in rock-type raw volume abundance and relative proportion of cumulative volume over Earth's history. (**A**) Igneous rock volumes show little variation throughout the Archean-Neoproterozoic but a rapid increase in the Phanerozoic. (**B**) Siliciclastic rock volumes show a steep increase from 3000 to 2400 Ma, no increase from 2400 to 600 Ma, and then rapidly increase across the Neoproterozoic-Phanerozoic boundary. (**C**) Carbonate volume increases linearly across most of Earth's history and rapidly across the Neoproterozoic-Phanerozoic boundary. (**D**) Metasedimentary rocks increase from 3000 to 2400 Ma, show no increase from 2400 to 600 Ma, and then increase sharply in the Phanerozoic before sharply decreasing toward the present day. (**E**) Proportion of sedimentary to igneous rocks in weatherable crust over time, showing increase in all sedimentary rock types relative to igneous rocks from 3000 to 2400 Ma, no trend from 2200 to 600 Ma, and a second sharp increase from 600 to 400 Ma. The fraction of cumulative volume of units at a given point in time (t) is calculated for units of given rock type (R_i, km^3) within preserved weatherable crust as $\frac{\sum R_i\ (\text{age}\geq t)}{\sum R_i\ (\text{age}\geq t)+\ldots\sum R_j\ (\text{age}\geq t)}$. One SD error (inherited from uncertainty in the age and volume of individual units) is shaded with hatched fill [cross-hatched in (E)]. Mean values are plotted in darker shade.

A second episode of relative sediment accumulation across the Neoproterozoic-Phanerozoic boundary (600 to 400 Ma) is also evident from these results. Sediment proportion increases by ~20% in this interval (Fig. 1E; comparing values at 400 Ma to those at 600 Ma), coincident with the formation of the Great Unconformity. The Great Unconformity is a global palaeogeomorphic surface, plausibly formed by massive erosion, characterized by the widespread juxtaposition of ancient igneous and metamorphic crystalline basement rocks with relatively undeformed and comparatively young sedimentary cover (*23*–*25*). Even if apparent changes in the lithological character of preserved crust across the Great Unconformity is an artifact related to preferential sediment removal, the lithochemical composition of preserved ancient crust can only have been biased by this event toward an igneous end-member composition by ~20%. We therefore conclude that the rock record is a viable candidate archive of crustal lithochemical evolution.

Overall, preserved rock abundances (Fig. 1E) do not support a widespread preservation bias against sedimentary rocks (*11*, *26*). Our results show that lithological proportions of cumulative rock volumes have been static over most of Earth history (around 2400 to 600 Ma; Fig. 1E), i.e., lithological equilibrium. We interpret lithological equilibrium to result from near-steady-state between the rates of accumulation and destruction of lithologies relative to one another. This result strongly suggests both that regional to continental erosion rates are limited by tectonics, rather than lithology (*27*, *28*), and that there is no substantial lithological bias induced by tectonic settings that more rapidly undergo exhumation.

We interpret the Macrostrat record of lithological proportions within preserved crust as being informative regarding the balance of rock type accumulation versus destruction over time. The lengthy period of lithological equilibrium from 2400 to 600 Ma represents near-steady-state between various forms of tectonic activity. That is, on the longest time scales, balance is observed between the rates of igneous, sedimentary, and metamorphic rock formation and destruction rates. Lithological equilibrium in the rock record is book ended by apparent changes in the rates at which sedimentary rocks were incorporated into long-term continental crustal reservoirs, across the Archean-Proterozoic and Proterozoic-Phanerozoic transitions (Fig. 1E). These episodes of change may represent shifting rates of relative accumulation or focused episodes of bias-inducing erosion. Regardless, lithological equilibrium throughout much of the rock record shows that preserved rocks represent a valuable sample of crustal lithochemical evolution.

The step changes in lithological proportions that we identify (Fig. 1E) may be partially or completely related to focused episodes of preservation bias during two exceptional episodes of erosion. For example, these could be driven by global glaciations that eroded deeply into continental crust, preferentially removing sedimentary rocks—perhaps due to their colocation in upper layers of crust. We discuss the relative merit of accumulation, destruction, and mixed scenarios in fig. S12. However, the least stringent mass balance constraints on interpreting the Macrostrat record of lithological and compositional change comes from the scenario that we consider in depth in the following sections: one where ancient lithological proportions are unbiased and lithological proportion changes record shifts in the Earth system.

Chemical evolution of weatherable continental crust

We explored the consequences of lithological evolution for the average chemical composition of weatherable continental crust, focusing on P. Alongside P, we reconstruct the compositional evolution of nickel (Ni), molybdenum (Mo), and the major elements. These have been considered by various previous studies of sedimentary and igneous rock P concentrations and bulk crustal chemistry over time (*3*, *29*), hence providing useful points of comparison for our approach. We calculate an average composition for all preserved rocks greater than a given age at each time step, therefore tracking long-term evolution rather than shorter-term variability.

Macrostrat presently has greatest data coverage in North America (*7*). It is therefore important to check that results from Macrostrat capture global and not solely regional signals. To test this, we compare against results from fine-grained sediments, which average out the compositional heterogeneity of their source terranes. By doing so, fine-grained sediments represent an alternative to volume weighting of discrete units (Macrostrat) when determining crustal compositional averages.

Our results from Macrostrat for the modern-day exposed crust are within error of previous estimates (fig. S2) (*30*). Sedimentary rock major element chemistry is broadly stable since 2000 Ma, comparing well to previous estimates obtained using the global-scale Sedimentary Geochemistry and Paleoenvironments Project (SGP) database (figs. S1 to S3) (*4*). Protolith compositions obtained following the unweathering procedure of Lipp *et al.* (*4*, *31*) compare well between SGP and both sediment-only and mixed (including igneous rock) results from Macrostrat (figs. S2 and S3).

These observations suggest that global resurfacing (e.g., formation of the Great Unconformity) has not led to a large discrepancy between the major element compositions of preserved crust and ancient emergent crust. We therefore are able to extract a reliable record of crustal P concentrations from Macrostrat, ruling out that any possible lithological biasing of ancient crustal segments has consequently biased average elemental compositions.

We find that weatherable crust concentrations of P are stable between 3000 and 1100 Ma (Fig. 2B), being within error of previous studies (*3*). However, the relative accumulation and increasingly P-rich composition of sedimentary rocks then acted to decouple the weatherable crust P concentration from that initially set by igneous processes (*1*, *3*), resulting in a threefold increase across the Neoproterozoic-Phanerozoic transition (600 to 400 Ma; Fig. 2B). This result is in stark contrast to several previous studies. Specifically, our records of crustal P content diverge from those of Greber *et al.* (*3*) and Condie (*5*) from 1100 Ma onward (Fig. 2B), moving to higher values while those of Greber and Condie remain flat.

Greber *et al.* (*3*) used a two-component igneous mixing model calibrated to the isotopic composition of shales to estimate crustal P concentrations. This model neglects any distinct sedimentary contribution to crustal P reservoirs and thus remains constant into the phanerozoic, where our record instead captures large sediment-driven increases in crustal P (*4*, *5*). Condie (*4*, *5*) also does not identify the Phanerozoic increase in P present in our results due to intentionally excluding the abundant sedimentary reservoir found outside the shield settings. This outcome reveals the importance of tracking multiple components of crustal lithochemical evolution, as the biosphere may preferentially sequester nutrients within continental marginal sediments (*32*), while nutrient-poor distal sediments go on to be subducted.

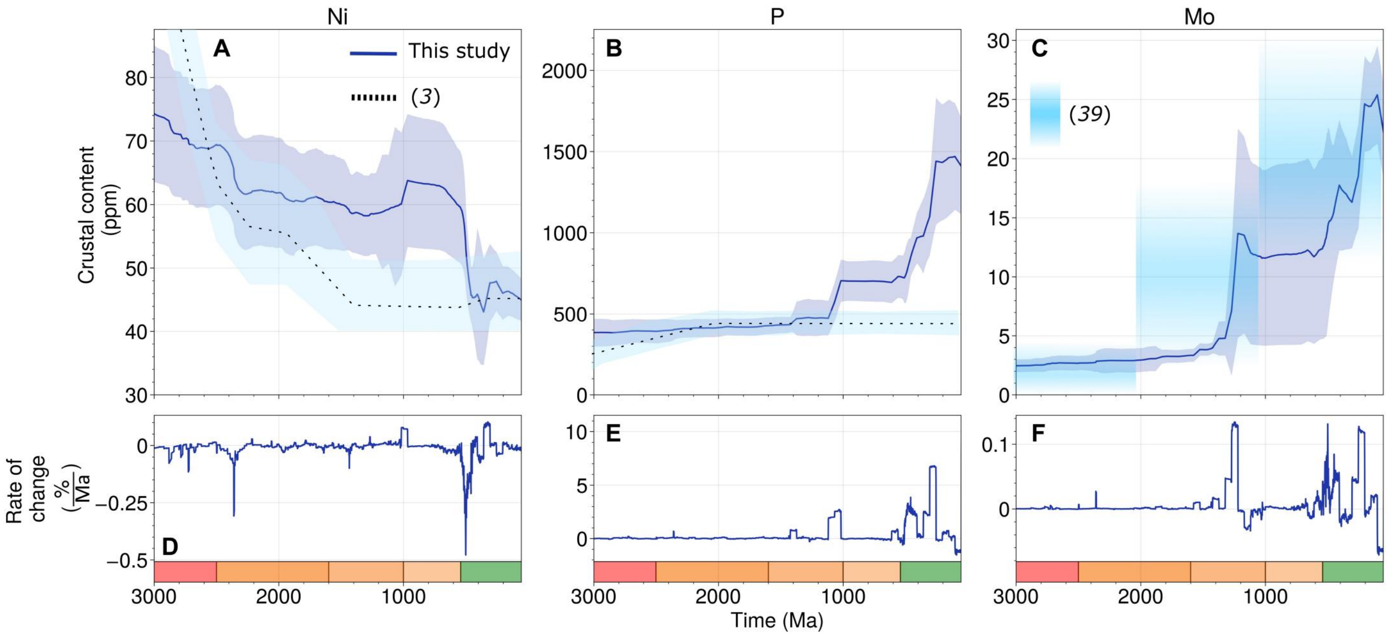

Fig. 2. Nutrient concentrations of average weatherable crust over Earth's history. (**A**) Nickel contents decline sharply in the end Archean due to the disappearance of Ni-rich komatiite and rise of Ni-poor sediments and igneous rocks. (**B**) Phosphorus concentrations are stable across the Archean-Proterozoic boundary but increase sharply in the Neoproterozoic-Phanerozoic. (**C**) Molybdenum concentrations increase during sediment accumulation but only after progressive deep ocean oxygenation. (**D** to **F**) Rates of change in crustal composition for each element. SE uncertainties from resampling are shaded in (A) to (C).

In another case, our results help to resolve discrepancies between previous studies of ancient P cycling, namely, disagreement on the robustness of apparent increases in sedimentary P content across the Neoproterozoic-Phanerozoic boundary (*33*). Reinhard *et al.* (*32*) suggest a Neoproterozoic increase in siliciclastic P content related to increased marginal P-burial and oxidant-driven P recycling. However, Laakso *et al.* (*14*) demonstrated that, considering the skew introduced by P-enriched outlier sediments, there is no statistically meaningful increase in mean sedimentary P content throughout the Neoproterozoic-Phanerozoic.

Our results directly account for volumetrically small but P-rich units. Siliciclastic P content increases abruptly compared to Archean-Mesoproterozoic units in the Neoproterozoic. Phosphorus concentrations in weatherable crust then approach a Phanerozoic equilibrium that is twofold higher than the Precambrian average (Fig. 2B). Therefore, while there is no obvious trend in sedimentary P content since the Neoproterozoic (*14*), higher P concentration in Neoproterozoic and younger sediments compared to earlier Archean-Mesoproterozoic sediments (*32*) is evident and does not simply represent overweighting of outliers.

Ni concentrations decline in our results as in Greber *et al.* (*3*) and other previous studies (*34*, *35*), being within two SE throughout Earth's history (Fig. 2A). The increasing fraction of sediment across 2400 Ma does not affect weatherable crust Mo concentrations substantially (Fig. 2C), an outcome supported by upper crustal Mo concentrations in contemporaneous sediments (*30*) (Fig. 2C). The Mesoproterozoic and Neoproterozoic-Phanerozoic step increases in weatherable crust Mo content that we recover are consistent with increased Mo mobilization during enhanced oxidative weathering of the continents (*36*–*39*).

Overall, results from Macrostrat indicate that the history of P enrichment in the crust is idiosyncratic compared to other nutrients: Our record reproduces those compiled previously for Ni and Mo (Fig. 2, A and C) but is divergent for P (Fig. 2B). One plausible explanation for this is increasingly efficient recycling of P by life from deep to shallow sedimentary environments, leading to enhanced P burial in marginal sediments over time (*40*–*42*). Our observations indicate that evolving marginal marine sediment compositions did not merely passively record changing biogeochemical dynamics. Rather, the incorporation of these marginal sediments into continental crust also reshaped the nutrient profile of weatherable crust.

The dramatic changes in P content in our results from 600 to 400 Ma occur coeval with rapid changes in raw volume of preserved rock (the Great Unconformity) and the relative abundance of sedimentary rock within weatherable crust. We now ask whether it is physically plausible for such a rapid shift in the lithochemical nature of weatherable crust to take place, at all, and specifically at this time in Earth's history.

Geological requirements for rapid crustal phosphorus enrichment

Rapid change in the average P concentration of weatherable crust may be achieved by the destruction of old rocks during weathering and the accumulation of compositionally distinct younger sediments; in other words, winnowing of uppermost crustal volume by erosion coupled to retention of P within preserved marginal sediments at a higher concentration than in bulk crust. Total crustal mass balance is balanced in this scenario by addition of fresh igneous rock to the crust, ultimately derived from the mantle. We test the geodynamic plausibility of this mechanism by considering mass balance constraints (see Materials and Methods for further

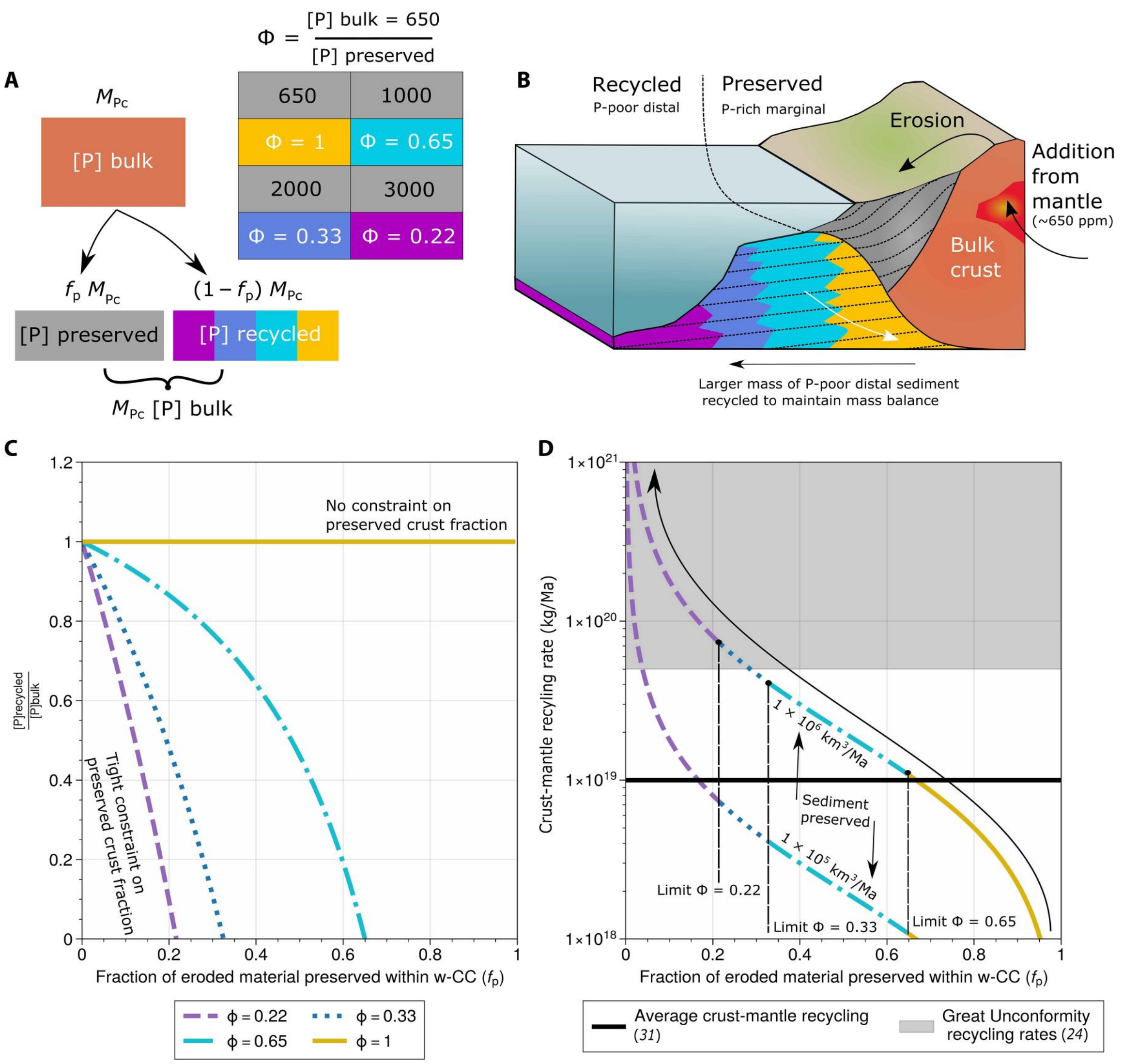

Fig. 3. Mass balance constraints on an evolving P concentration of weatherable continental crust. (**A**) Formulation of mass balance calculation, which considers crust-mantle recycling rates and P content of subducted sediments. (**B**) Schematic illustration of the mass balance test performed, showing the volume of subducted crust needed to satisfy mass balance for two contrasting P ratios between preserved crust—for which Macrostrat delivers observed values over time, with some uncertainty—and subducted material. (**C**) Ratio of [P] in recycled material relative to bulk crust (ϕ) imposes a constraint on the fraction of eroded material that may be preserved within weatherable crust (solution to Eq. 4; see Materials and Methods). Low values of ϕ, which correspond to strong P enrichments of preserved crust, demand high volumes of recycled material and thus low volumes of preserved material. w-CC, weatherable continental crust. (**D**) Crust-mantle recycling rates required across a range of values of ϕ. Each line is superimposed, with lines where ϕ = 1 extending the furthest. These values are compared to average crust-mantle recycling rates and those inferred across the Great Unconformity (*24*, *25*).

details; Fig. 3), namely, that sufficient P must be eroded and mobilized to become concentrated within the mass of preserved P-rich crust that is suggested by analysis of the Macrostrat database.

We consider the P content and mass of weathered Precambrian crust [P_{Pc}^{Bulk}, parts per million (ppm); M_{Pc}, kg], some portion of which is preserved (f_p; 0 to 1) as marginal sedimentary rock ($M_{Ph}^{Preserved}$) and the rest of which ($1 - f_p$) is ultimately recycled via subduction ($M_{Ph}^{Recycled}$; Fig. 3A). Phosphorus may be differentially partitioned between preserved and recycled material due to preferential biomass deposition on the continental shelf and hence nutrient burial within marginal sediments (Fig. 2B) (*32*). We describe the extent to which P was favorably partitioned into preserved sediment via a ratio, $\phi = P_{Pc}^{Bulk}/P_{Ph}^{Preserved}$. For a range of ϕ, chosen from our reconstructed crustal P content evolution (Fig. 2B), and the range of physically possible values for f_p, we calculate the P content (>0 ppm) of subducted material that satisfies mass balance (Fig. 3C) and then solve for crust-mantle recycling rate (Fig. 3D).

The largest and most rapid changes in weatherable crust P content (Fig. 2B) occur across the Neoproterozoic-Phanerozoic boundary, at 600 to 400 Ma. These are the most important changes to test for geodynamic plausibility, and our results for this time frame inform the values of ϕ explored in Fig. 3. The most extreme constraint from our observations is production of

preserved Phanerozoic sediment with 3000-ppm P from 650-ppm P bulk Precambrian crust coeval with formation of Phanerozoic igneous crust with <1000-ppm P and resulting in an average weatherable crust P concentration of ~1400 ppm. Comparing the results of our test to independent constraints on crust-mantle recycling rates (*4*, *24*, *25*, *31*), we find that altering weatherable crust P concentrations during 600 to 400 Ma by the reconstructed amount requires crust-mantle recycling above the geodynamic background rate (Fig. 3, B to D). This time interval happens to encompass the formation of the Great Unconformity (*43*–*45*). The occurrence Great Unconformity was therefore of great relevance for reworking and enriching the crustal P reservoir, denuding the continents of P-poor rock and producing in their stead P-rich sediments at a rate well above the background of Proterozoic tectonic and crust-mantle recycling activity (Fig. 3).

Crustal lithochemical evolution and the global phosphorus cycle

The dramatic step change in crustal P concentrations at 600 to 400 Ma may have had important implications for global P weathering fluxes and hence the long-term availability of P to life. In particular, increasing crustal P concentrations should drive larger P weathering fluxes independent of any change in the rate of overall chemical or physical crustal weathering. However, to evaluate whether or not crustal P enrichment is biogeochemically important, we must quantify the effect of changing crustal P concentrations (all else being equal) on the global P weathering flux.

We estimate the global weathering flux of P over time with a modified geological carbon cycle model (see Material and Methods, fig. S4, and text S1 for detailed caveats) (*43*, *44*). This model was originally applied to simulate the long-term inorganic carbon cycle by considering silicate and carbonate weathering feedbacks (*44*) and is successfully calibrated with the modern carbon cycle. We introduce the evolution of lithological P content (Fig. 2B) to the model and calculate the amount of P released per mole of CO_2 reacted during global carbonate and silicate weathering.

We track P weathering fluxes from both emergent and submerged crust (Fig. 4A), considering a scenario where P is released from emergent and submerged crust with equal efficiency and where no P is released during submerged crust weathering. In the former case, we find that lithochemical evolution acts to maintain global P weathering fluxes that are (within error) invariant from 3000 to 600 Ma (Fig. 4A), before increasing to levels not seen before in Earth's history during the Phanerozoic. In contrast, if submarine basalt weathering is assumed not to release bioavailable P (*45*), then the global P weathering flux may have increased substantially during continental emergence from 3000 to 2200 Ma (more rapid land emergence shifts this feature to earlier Earth history; see figs. S5 and S6).

In both cases, we find that P fluxes increase across the Neoproterozoic-Phanerozoic boundary (Fig. 4A), owing to the accumulation and subsequent reweathering of P-enriched sediments. If submerged crust weathering is permitted to contribute to the global P flux, then we find that the seafloor was the principal source of P for the biosphere in the Archean-Paleoproterozoic (Fig. 4B). A gradual decline in seafloor-sourced P and increase in continental-sourced P would have occurred as emergent crust became the principal sink for atmospheric carbon during weathering, accelerated sharply by (prescribed, in our calculations) oxygenation of the deep oceans that initiated the seafloor hydrothermal P sink (*46*–*48*) and (observed, in our results from Macrostrat) coeval enrichment of the continental P reservoir (Fig. 2B). This possibility points toward another mechanism for enriching the continental P reservoir: a shift in biomass deposition away from deep sea environments toward continental shelves as riverine P fluxes coming off the continents increase.

The sustained uptick in P weathering flux across the Neoproterozoic-Phanerozoic boundary (Fig. 4B) results in a predicted value for the present day that is the same within error as the observed modern flux (*49*). This result is notable, as the compositional constraints from ancient sedimentary whole-rock analyses are an input to the weathering model entirely independent of modern weathering fluxes. Our results suggests that our reconstruction of weatherable crust is a viable input to models of long-term biogeochemical evolution.

DISCUSSION

We have shown that preserved rocks represent a reliable archive of crustal lithochemical evolution. The rock record evidences an overall lack of preservation bias against sedimentary rocks (Fig. 1). By weighting geochemical measurements by the volume of rock units within Earth's weatherable crust, we remove issues associated with the overweighting of outliers in the calculation of average chemical compositions. We obtain a record of average crustal P concentration (Fig. 2B). This record resolves discrepancies between published analyses of fine-grained sedimentary and igneous rocks and reveals a threefold increase in the average crustal P concentration across the Neoproterozoic-Phanerozoic boundary.

If P is considered as the ultimate limiting nutrient for life (2), then global continental exhumation coupled to nutrient-rich sediment accumulation across the Neoproterozoic-Phanerozoic boundary was highly consequential for setting the total biomass subsequently permitted by continental subaerial weathering. These coupled changes were made possible by coeval removal of ancient rock and accumulation of young P-rich sediment during formation of the Great Unconformity (Fig. 3).

According to our results, global crustal P concentrations only increased substantially after the Neoproterozoic Oxygenation Event (fig. S11) (*48*) and were largely stable and comparatively low over earlier Earth history. This is a relevant finding, as steadily increasing crustal P concentrations have been previously linked to stepwise atmospheric oxygenation (*1*). The translation of these changes in sedimentary volume fraction and P concentration into changes in baseline P weathering flux may be further delayed, given substantial lag times between sedimentary rock formation and eventual exhumation (fig. S13). Our results suggest a plausible alternative mechanism, at least for Neoproterozoic oxygenation: enhanced rates of global erosion, which may have transiently increased P input into the oceans during the formation of the Great Unconformity. Macrostrat provides evidence of this large-scale crustal reworking at 600 to 400 Ma (Fig. 1), coeval with changes in ocean redox proxies (fig. S11).

In order for a global erosive event to impact global productivity or oxygen production, P mobilized physically during erosion would also need to be made available to life by chemical weathering. This

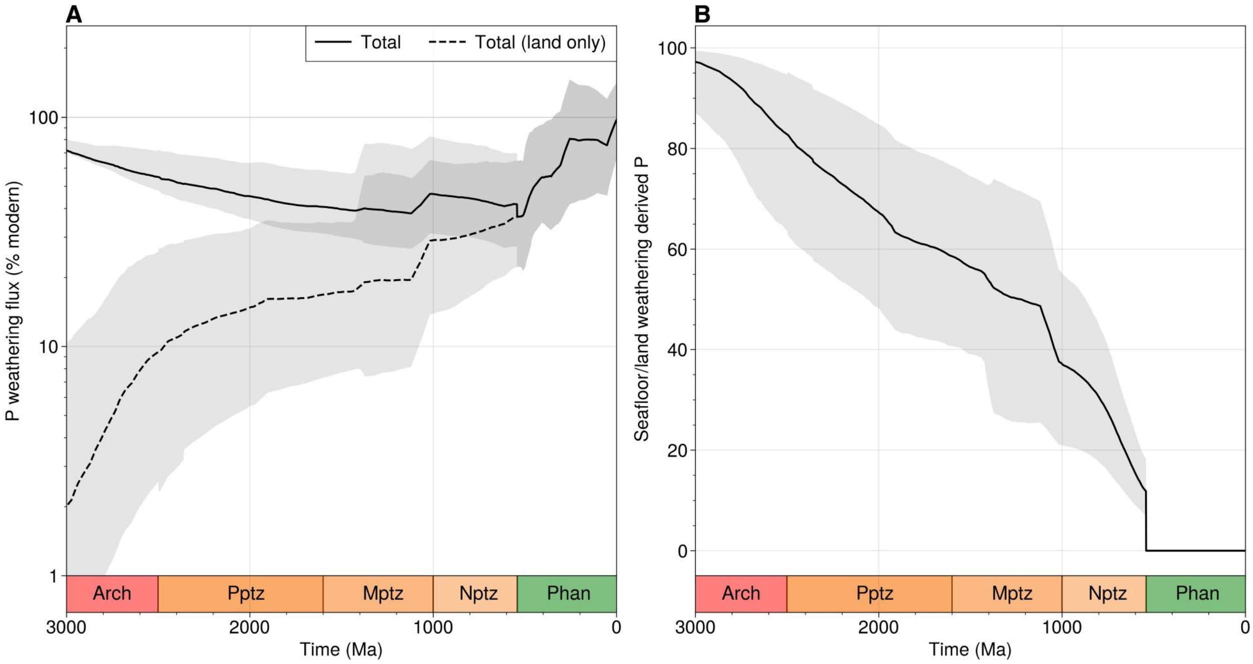

Fig. 4. Consequences of weatherable crust evolution for the P cycle. (**A**) Phosphorus fluxes relative to modern day (*49*) over Earth's history, given land emergence rate of Flament *et al.* (*58*). See fig. S5 and S6 for a rapid land emergence scenario. (**B**) Total P fluxes for land only and land plus seafloor scenarios. If seafloor weathering is assumed not to have contributed bioavailable P to the oceans, then P fluxes may have increased by up to an order of magnitude from 3000 to 2000 Ma. Otherwise and at least from 2000 Ma onward, P fluxes are stable until the Cambrian boundary, after which a step increase occurs.

step requires a coeval perturbation to the carbon cycle, for which there is some evidence in the Neoproterozoic (*48*, *50*, *51*). Notable perturbations to the carbon cycle are unsustainable in the long term, due to stabilizing negative feedbacks (fig. S4), but may trigger mechanisms that go on to permanently enrich the crust in nutrients, e.g., enhanced oxidative P recycling from deep to marginal settings coupled to subsequent retention on continental shelves and/or high sedimentation rates that enhanced marginal organic C and P burial (*42*, *52*–*54*).

We conclude that changes in the average P concentration of weatherable crust acted as a positive feedback on global P availability to life but is unlikely to have triggered Archean or Neoproterozoic step increases in atmospheric oxygen content. Continental retention of increasingly P-rich marginal sediments represents a mechanism to enhance global primary productivity via crustal P enrichment, enabled by increasingly efficient P recycling (*42*), a possible shift in the locus of overall crustal weathering and biomass deposition to continents and continental shelves (Fig. 4, A and B), and the erosion of ancient P-poor crustal rocks (Fig. 3). An implication of this mechanism is a consequently lower flux of P being recycled into the mantle, which is tentatively supported in the data compilation of potentially genetically related sedimentary and volcanic rocks published by Ma *et al.* (*55*).

Our results have greatest spatial coverage in North America. Therefore, despite mass balance arguments for global signatures being recorded in Macrostrat, we cannot presently rule out that a large portion of the signal in our data derives from regional tectonic history. Considering a worst case scenario for the relevance of the crustal P-enriching mechanisms that we have discussed (fig. S12), the entirety of the apparent increase in sedimentary fraction across the Neoproterozoic-Phanerozoic transition could reflect a focused episode of regional erosion that was unique to North America. However, the potentially redox-dependent shift in sedimentary P at this time does appear to be global (*32*). Hence, while weatherable crust beyond that represented in Macrostrat may not show the same inflection in fraction of sedimentary rock — and especially young sedimentary rock—crustal P concentrations will still increase after this point in time, albeit less rapidly than in the scenario where Macrostrat is globally representative.

Our results speak to the overall composition of preserved crust as sampled by Macrostrat, rather than the exact temporal structure of crustal weathering. Hence, our results do not constrain the possible biogeochemical relevance of massive but short-lived P enrichment of the crust, achieved by emplacement of igneous rocks with relatively high P contents that were then rapidly destroyed by weathering, e.g., large igneous provinces (LIPs). However, our results do show that the removal of P-poor sedimentary rock and retention of P-rich sedimentary rock is more important for modulating the global P weathering flux over long time scales and large spatial scales than the discrete emplacement of P-rich LIPs. Ultimately, LIPs are not sufficiently voluminous or P-rich to substantially modify the average P concentration of average weatherable crust. Adding 6 million km^3 of P-rich mafic rock with P concentration of 800 ppm (*56*) to the volume of weatherable crust sampled in Macrostrat (2.6×10^8 km^3; Archean-Proterozoic baseline P concentration of 500 ppm) would result in only a 2.4% relative increase in its average P concentration. This contrasts with the threefold increase driven by P-rich sediment retention in our results.

Assessing the timing and tempo of global biogeochemical change more broadly will require evaluating the following multiple

driving factors: changes in accommodation space, volcanic outgassing rate, topography, paleogeographic distribution of land, continental freeboard, average crustal nutrient concentration, biological innovation, and much more. Doing so will require simultaneous evaluation with falsifiable biogeochemical models (*57*). Independent of which factors are most important for driving biogeochemical change, the crucial insights from our work remain: that preserved rocks represent a remarkably robust archive of crustal evolution and that global erosion coupled to continental nutrient retention rapidly enriched weatherable crust in P at the dawn of the Phanerozoic.

MATERIALS AND METHODS

Data retrieval and handling

The Macrostrat database is organized into stratigraphic columns with a geographic footprint, each of which is associated with unique units (*10*). Macrostrat "columns"—the dataset used in our analysis—are constructed from a combination of surface-exposed geology and subsurface information. Thus, Macrostrat columns typically reflect both the general geology of the surface and the subsurface down to the deepest known rock units in each region. Hence, they provide some measure of crustal geology that is more inclusive than estimates that are based only on geological maps. Rock units recognized on geological maps that span multiple columns will appear as distinct units within each of those columns. All data are obtained with calls to the Macrostrat application program interface (API), e.g., as follows: https://macrostrat.org/api.

Rock volumes were tabulated by multiplying unit thicknesses by areal extent and dividing this total volume linearly across the reported temporal extent of the unit, i.e., time between maximum and minimum age, rather than a best estimated age with some uncertainty. This age model is currently the most self-consistent approach available in Macrostrat. An obvious caveat is that, while sediments are laid down continuously, large volume igneous rocks may form discretely. However, our approach describes well the uncertain formation times of more ancient igneous rocks and removes any methodological bias from the present result.

We obtain unit age-volume distributions for igneous, sedimentary, and metasedimentary rocks, respectively. Each underlying data distribution was randomly resampled with replacement 1000 times, weighting for unit volume, to generate uncertainties. This approach to uncertainty estimation is reasonable on several counts. We are interested in our approach in calculating the total preserved volume of rock from iteratively larger spans of Earth history. Hence, it is necessary to estimate the total volume of units from a given time span, rather than the spread of data or mean. Macrostrat cannot and does not perfectly represent subsurface rock geometry, yet constraints are limited at present on the uncertainty associated with individual unit volumes. We must therefore use an alternative means to uncertainty estimation.

Resampling with replacement in effect estimates the resulting suite of total volumes per unit time that might be obtained if the underlying data were collected several times over, i.e., in our case, if the Earth's surface were mapped and digitally represented with a database (Macrostrat) repeatedly, each time returning a slightly different result. This offers insight into how sensitive derivative results from Macrostrat are to the presence or absence of, e.g., particularly volumetrically large individual units.

Compositional whole-rock data measurements were obtained via the Macrostrat API for each unit. Measurements were averaged together where multiple values were reported for the same unit. The resulting unit compositions were used to calculate grand average compositions of weatherable crust. Each contributing composition was weighted in this calculation by the volume contribution of the parent unit to the total volume, in each 1-Ma time step. Where data were sparse (number of parental unit average composition < 3), the last successfully retrieved average composition was propagated through the calculation. This condition was met principally during reconstruction of Archean sedimentary crustal compositions.

Standard error uncertainties on arithmetic mean compositions were obtained using these data ($\frac{\sigma}{\sqrt{n}}$) and propagated analytically alongside the error in lithological relative abundance when calculating the average weatherable crust compositions. All major element—but not minor element—compositions, including reference values, are reported after being normalized to 100 weight % within the system Si + Al + Fe + Mg + Na + Ca + K (standard oxides). We calculate the likely protoliths for sediments by projecting them parallel to a weathering trend on to an array of pristine igneous rocks (*4*).

Phosphorus weathering model

The phosphorus weathering flux was estimated using the geological carbon cycle model of Krissansen-Totton *et al.* (*44*), with some modifications for the quantification of P cycle (*43*). The revised code is available at Github (https://github.com/bronzitte/Early_earth_cycle.git). First, we replaced the input factor of continental growth history used in the original code by the reconstructions for the emerged area of subaerial land, including the slow emergence case (fig. S7A) (*58*) and fast emergence case (fig. S7B) (*59*). Second, we set the vegetation compensating factor (VCF) as 0.25 (*43*) rather than 0.1 in the original code for Eq. 1 (fig. S8) (*44*)

$$F_{\text{bio}} = 1 - \frac{1}{\frac{1}{1-\text{VCF}} + e^{-10\times\frac{t-0.6*10^9}{10^9}}} \quad (1)$$

where F_{bio} accounts for vegetation effect on continental weathering and t is geological time in years. We kept all other parameters consistent with the original code. The Monte Carlo simulation has been tested for 1000, 5000, and 10,000 runs, and the results are identical.

Our weathering simulations calculate the global fluxes (mole C consumed per year) of continental silicate weathering (FC_{silicate}), continental carbonate weathering ($FC_{\text{carbonate}}$), and seafloor silicate weathering (FS_{silicate}) (figs. S9 and S10), which are all well calibrated by the modern weathering fluxes (*44*). We further incorporated the evolution of phosphorus chemistry (P content and distribution in different reservoirs) in continental crust (Fig. 2B) into the model to simulate the weathering flux of P, sampling the error envelopes of these inputs randomly alongside all others. P, speciated principally as orthophosphate (PO_4^{3-}), is mainly present as impurity or substitution of CO_3 and SiO_4 in carbonate (*60*) and seafloor basalt (*61*), respectively, but as apatite minerals in continental silicate rocks (*61*). Accordingly, we estimated the P-releasing fluxes by separately considering these sources:

1) Phosphorus released during continental carbonate weathering was calculated by multiplying the P content in carbonate rock

($[P]_{carb}$, ppm) by $FC_{carbonate}$ (figs. S9B and S10B) and the unit conversion factors.

2) Phosphorus released during continental silicate (igneous + siliciclastic) weathering was calculated by multiplying P content in silicate rock ($[P]_{silicate}$, ppm) by $FC_{silicate}$ (figs. S9A and S10A) and the unit conversion factors. It is observed that global silicate weathering flux (g/year) has a linear correlation with CO_2 consumption flux (mole/year): 1 mole CO_2/year = 100 g silicate/year (*62*), which is used here to convert the units. Here, we assume a congruent release of P during silicate weathering.

3) Seafloor weathering is a net sink rather than source for P in the modern oxidizing ocean due to the adsorption of phosphate onto ferrihydrite (*47*) and other secondary minerals. Recently, Syverson *et al.* (*45*) proposed seafloor weathering as a supply of P to the anoxic and ferruginous ocean on early Earth. Here, we estimated the potential supply of P from seafloor weathering to the early ocean. The P weathering flux was calculated by multiplying $FS_{silicate}$ (figs. S9C and S10C) by the P content of seafloor silicates (assumed to be 500 ppm in this study). However, after the oxygenation of the ocean at 0.5 Ga, the weathering flux was set to be 0 due to expected widespread adsorption of P onto ferrihydrite.

We designed a second Monte Carlo simulation to propagate error through the weathering model derived from rock volume/compositional uncertainties. Each simulation randomly selects one set of values from the weathering model data and volume/concentration uncertainty ranges. Results of 500 runs and above are identical.

Mass balance calculation

We tested whether the changes in minor element chemistry in our reconstructed history of crustal chemical and lithological evolution are externally consistent, i.e., physically plausible, by considering mass balance. We focus on the most dramatic changes in the record, which occur across the Neoproterozoic-Phanerozoic boundary.

Erosion of some mass of Precambrian crust (M_{Pc}, kg) will result in a mass of Phanerozoic sediment, some fraction of which will be preserved ($M_{Ph}^{Preserved}$) and the rest of which will be recycled into the mantle via subduction ($M_{Ph}^{Recycled}$). A mass balance relationship exists for these variables

$$M_{Pc} = M_{Ph}^{Preserved} + M_{Ph}^{Recycled} \quad (2)$$

P with some concentration (P, ppm) derived from M_{Pc} must be distributed between the mass fraction of preserved sediment (f_p)

$$f_p = \frac{M_{Ph}^{Preserved}}{M_{Pc}} \quad (3)$$

and the mass fraction of recycled material

$$1 - f_p = \frac{M_{Ph}^{Recycled}}{M_{Pc}} \quad (4)$$

The mass of P must be equal in this formulation between that obtained from eroded Precambrian crust and the sum of preserved and subducted material, such that

$$f_p \; P_{Ph}^{Preserved} + (1 - f_p) \; P_{Ph}^{Recycled} = P_{Pc}^{Bulk} \quad (5)$$

Rearranging and simplifying Eq. 5 to solve for $P_{Ph}^{Recycled}$

$$P_{Ph}^{Recycled} = \frac{P_{Pc}^{Bulk} - f_p \; P_{Ph}^{Preserved}}{(1 - f_p)} \quad (6)$$

On the basis of our results for crustal compositional scenarios, we test various scenarios for rates of change in crustal P content. We can describe the extent to which P was favorably partitioned into preserved sediment via a ratio, $\phi = P_{Pc}^{Bulk} / P_{Ph}^{Preserved}$. For a range of values for ϕ, we can substitute bulk Precambrian and preserved Phanerozoic crustal compositions into Eq. 5 and explore the compositional range of $P_{Ph}^{Preserved}$ and f_p that satisfies mass balance within physical limits (P content > 0 ppm; $f_p > 0$). Rearranging Eq. 4, we can then solve for the range of corresponding necessary masses of recycled crust. We upscale the volumes of preserved crust and eroded material by 6.5-fold, consistent with Macrostrat presently covering <20% of the global continental crust.

Correction (21 July 2023): The original Figs. 1 and 3 included incorrect labels. The y axis scales in Fig. 1 (A to D) were incorrect and have been updated. The arrows in Fig. 3D were incorrectly labeled using the unit "years" instead of "Ma" (million years). The PDF and HTML have been corrected.

Supplementary Materials

This PDF file includes:
Sections S1 to S3
Figs. S1 to S13
References

REFERENCES AND NOTES

1. G. M. Cox, T. W. Lyons, R. N. Mitchell, D. Hasterok, M. Gard, Linking the rise of atmospheric oxygen to growth in the continental phosphorus inventory. *Earth Planet. Sci. Lett.* **489**, 28–36 (2018).
2. K. J. Zahnle, D. C. Catling, M. W. Claire, The rise of oxygen and the hydrogen hourglass. *Chem. Geol.* **362**, 26–34 (2013).
3. N. D. Greber, N. Dauphas, A. Bekker, M. P. Ptáček, I. N. Bindeman, A. Hofmann, Titanium isotopic evidence for felsic crust and plate tectonics 3.5 billion years ago. *Science* **357**, 1271–1274 (2017).
4. A. G. Lipp, O. Shorttle, E. A. Sperling, J. J. Brocks, D. B. Cole, P. W. Crockford, L. D. Mouro, K. Dewing, S. Q. Dornbos, J. F. Emmings, U. C. Farrell, A. Jarrett, B. W. Johnson, P. Kabanov, C. B. Keller, M. Kunzmann, A. J. Miller, N. T. Mills, B. O'Connell, S. E. Peters, N. J. Planavsky, S. R. Ritzer, S. D. Schoepfer, P. R. Wilby, J. Yang, The composition and weathering of the continents over geologic time. *Geochem. Perspect. Lett.* **7**, 21–26 (2021).
5. K. C. Condie, Chemical composition and evolution of the upper continental crust: Contrasting results from surface samples and shales. *Chem. Geol.* **104**, 1–37 (1993).
6. G. J. S. Bluth, L. R. Kump, Phanerozoic paleogeology. *Am. J. Sci.* **291**, 284–308 (1991).
7. S. E. Peters, C. R. Walton, J. M. Husson, D. P. Quinn, O. Shorttle, C. B. Keller, R. R. Gaines, Igneous rock area and age in continental crust. *Geology* **49**, 1235–1239 (2021).
8. E. E. Stüeken, R. Buick, R. E. Anderson, J. A. Baross, N. J. Planavsky, T. W. Lyons, Environmental niches and metabolic diversity in Neoarchean lakes. *Geobiology* **15**, 767–783 (2017).
9. J. M. Husson, S. E. Peters, Atmospheric oxygenation driven by unsteady growth of the continental sedimentary reservoir. *Earth Planet. Sci. Lett.* **460**, 68–75 (2017).
10. S. E. Peters, J. M. Husson, J. Czaplewski, Macrostrat: A platform for geological data integration and deep-time earth crust research. *Geochem. Geophys. Geosystems* **19**, 1393–1409 (2018).
11. J. M. Husson, S. E. Peters, Nature of the sedimentary rock record and its implications for Earth system evolution. *Emerg. Top Life Sci.* **2**, 125–136 (2018).
12. S. E. Peters, J. M. Husson, Sediment cycling on continental and oceanic crust. *Geology* **45**, 323–326 (2017).
13. C. P. Slomp, P. V. Cappellen, The global marine phosphorus cycle: Sensitivity to oceanic circulation. *Biogeosciences* **4**, 10.5194/bgd-3-1587-2006, (2007).

14. T. A. Laakso, E. A. Sperling, D. T. Johnston, A. H. Knoll, Ediacaran reorganization of the marine phosphorus cycle. *Proc. Natl. Acad. Sci. U.S.A.* **117**, 11961–11967 (2020).
15. N. Moosdorf, S. Cohen, C. von Hagke, A global erodibility index to represent sediment production potential of different rock types. *Appl. Geogr.* **101**, 36–44 (2018).
16. B. Campforts, V. Vanacker, F. Herman, M. Vanmaercke, W. Schwanghart, G. E. Tenorio, P. Willems, G. Govers, Parameterization of river incision models requires accounting for environmental heterogeneity: Insights from the tropical Andes. *Earth Surf. Dynam.* **8**, 447–470 (2020).
17. A. M. Mloszewska, S. J. Mojzsis, E. Pecoits, D. Papineau, N. Dauphas, K. O. Konhauser, Chemical sedimentary protoliths in the >3.75Ga Nuvvuagittuq Supracrustal Belt (Québec, Canada). *Gondw. Res.* **23**, 574–594 (2013).
18. J. E. J. Martini, A late Archaean-Palaeoproterozoic (2.6 Ga) palaeosol on ultramafics in the Eastern Transvaal, South Africa. *Precambrian Res.* **67**, 159–180 (1994).
19. B. Ausín, E. Bruni, N. Haghipour, C. Welte, S. M. Bernasconi, T. I. Eglinton, Controls on the abundance, provenance and age of organic carbon buried in continental margin sediments. *Earth Planet. Sci. Lett.* **558**, 116759 (2021).
20. J. F. Read, Carbonate platforms of passive (extensional) continental margins: Types, characteristics and evolution. *Tectonophysics* **81**, 195–212 (1982).
21. M. Tang, X. Chu, J. Hao, B. Shen, Orogenic quiescence in Earth's middle age. *Science* **371**, 728–731 (2021).
22. P. Chowdhury, J. A. Mulder, P. A. Cawood, S. Bhattacharjee, S. Roy, A. N. Wainwright, O. Nebel, S. Mukherjee, Magmatic thickening of crust in non–plate tectonic settings initiated the subaerial rise of Earth's first continents 3.3 to 3.2 billion years ago. *Proc. Natl. Acad. Sci. U.S.A.* **118**, e2105746118 (2021).
23. S. E. Peters, R. R. Gaines, Formation of the 'Great Unconformity' as a trigger for the Cambrian explosion. *Nature* **484**, 363–366 (2012).
24. C. B. Keller, J. M. Husson, R. N. Mitchell, W. F. Bottke, T. M. Gernon, P. Boehnke, E. A. Bell, N. L. Swanson-Hysell, S. E. Peters, Neoproterozoic glacial origin of the Great Unconformity. *Proc. Natl. Acad. Sci. U.S.A.* **116**, 1136–1145 (2018).
25. K. T. McDannell, C. B. Keller, W. R. Guenthner, P. K. Zeitler, D. L. Shuster, Thermochronologic constraints on the origin of the Great Unconformity. *Proc. Natl. Acad. Sci. U.S.A.* **119**, e2118682119 (2022).
26. A. B. Ronov, V. E. Khain, A. N. Balukhovsky, K. B. Seslavinsky, Quantitative analysis of Phanerozoic sedimentation. *Sediment. Geol.* **25**, 311–325 (1980).
27. G. G. Roberts, N. White, B. H. Lodhia, The generation and scaling of longitudinal river profiles. *J Geophys. Res Earth Surf.* **124**, 137–153 (2019).
28. J. Veizer, F. T. Mackenzie, Treatise on Geochemistry, 369–407 (2003).
29. M. Tang, K. Chen, R. L. Rudnick, Archean upper crust transition from mafic to felsic marks the onset of plate tectonics. *Science* **351**, 372–375 (2016).
30. R. L. Rudnick, S. Gao, Composition of the Continental *Crust. Geochemistry* **3**, 659 (2014).
31. A. G. Lipp, O. Shorttle, F. Syvret, G. G. Roberts, Major element composition of sediments in terms of weathering and provenance: Implications for crustal recycling. *Geochem. Geophys. Geosystems* **21**, e2019GC008758 (2020).
32. C. T. Reinhard, N. J. Planavsky, B. C. Gill, K. Ozaki, L. J. Robbins, T. W. Lyons, W. W. Fischer, C. Wang, D. B. Cole, K. O. Konhauser, Evolution of the global phosphorus cycle. *Nature* **541**, 386–389 (2017).
33. N. J. Planavsky, D. Asael, A. D. Rooney, L. J. Robbins, B. C. Gill, C. M. Dehler, D. B. Cole, S. M. Porter, G. D. Love, K. O. Konhauser, C. T. Reinhard, A sedimentary record of the evolution of the global marine phosphorus cycle. *Geobiology* **21**, 168–174 (2023).
34. K. O. Konhauser, E. Pecoits, S. V. Lalonde, D. Papineau, E. G. Nisbet, M. E. Barley, N. T. Arndt, K. Zahnle, B. S. Kamber, Oceanic nickel depletion and a methanogen famine before the Great Oxidation Event. *Nature* **458**, 750–753 (2009).
35. K. O. Konhauser, L. J. Robbins, E. Pecoits, C. Peacock, A. Kappler, S. V. Lalonde, The Archean nickel famine revisited. *Astrobiology* **15**, 804–815 (2015).
36. E. E. Stüeken, D. C. Catling, R. Buick, Contributions to late Archaean sulphur cycling by life on land. *Nat. Geosci.* **5**, 722–725 (2012).
37. N. J. Planavsky, D. Asael, A. Hofmann, C. T. Reinhard, S. V. Lalonde, A. Knudsen, X. Wang, F. O. Ossa, E. Pecoits, A. J. B. Smith, N. J. Beukes, A. Bekker, T. M. Johnson, K. O. Konhauser, T. W. Lyons, O. J. Rouxel, Evidence for oxygenic photosynthesis half a billion years before the Great Oxidation Event. *Nat. Geosci.* **7**, 283–286 (2014).
38. A. C. Johnson, C. M. Ostrander, S. J. Romaniello, C. T. Reinhard, A. T. Greaney, T. W. Lyons, A. D. Anbar, Reconciling evidence of oxidative weathering and atmospheric anoxia on Archean Earth. *Sci. Adv.* **7**, eabj0108 (2021).
39. M. Thoby, K. O. Konhauser, P. W. Fralick, W. Altermann, P. T. Visscher, S. V. Lalonde, Global importance of oxic molybdenum sinks prior to 2.6 Ga revealed by the Mo isotope composition of Precambrian carbonates. *Geology* **47**, 559–562 (2019).
40. A. Paytan, K. McLaughlin, The oceanic phosphorus cycle. *Chem. Rev.* **107**, 563–576 (2007).
41. J. S. Compton, E. W. Bergh, Phosphorite deposits on the Namibian shelf. *Mar. Geol.* **380**, 290–314 (2016).
42. M. A. Kipp, E. E. Stüeken, Biomass recycling and Earth's early phosphorus cycle. *Sci. Adv.* **3**, eaao4795 (2017).
43. J. Hao, A. H. Knoll, F. Huang, R. M. Hazen, I. Daniel, Cycling phosphorus on the archean earth: Part I. Continental weathering and riverine transport of phosphorus. *Geochim. Cosmochim. Acta* **273**, 70–84 (2020).
44. J. Krissansen-Totton, G. N. Arney, D. C. Catling, Constraining the climate and ocean pH of the early Earth with a geological carbon cycle model. *Proc. Natl. Acad. Sci. U.S.A.* **115**, 4105–4110 (2018).
45. D. D. Syverson, C. T. Reinhard, T. T. Isson, C. J. Holstege, J. A. R. Katchinoff, B. M. Tutolo, B. Etschmann, J. Brugger, N. J. Planavsky, Nutrient supply to planetary biospheres from anoxic weathering of mafic oceanic crust. *Geophys. Res. Lett.* **48**, e2021GL094442 (2021).
46. C. G. Wheat, J. McManus, M. J. Mottl, E. Giambalvo, Oceanic phosphorus imbalance: Magnitude of the mid-ocean ridge flank hydrothermal sink. *Geophys. Res. Lett.* **30**, 10.1029/2003GL017318, (2003).
47. C. G. Wheat, R. A. Feely, M. J. Mottl, Phosphate removal by oceanic hydrothermal processes: An update of the phosphorus budget in the oceans. *Geochim. Cosmochim. Acta* **60**, 3593–3608 (1996).
48. R. Tostevin, B. J. W. Mills, Reconciling proxy records and models of Earth's oxygenation during the Neoproterozoic and Palaeozoic. *Interface Focus* **10**, 20190137 (2020).
49. J. Hartmann, N. Moosdorf, R. Lauerwald, M. Hinderer, A. J. West, Global chemical weathering and associated P-release — The role of lithology, temperature and soil properties. *Chem. Geol.* **363**, 145–163 (2014).
50. J. J. Williams, B. J. W. Mills, T. M. Lenton, A tectonically driven Ediacaran oxygenation event. *Nat. Commun.* **10**, 2690 (2019).
51. G. A. Shields, B. J. W. Mills, M. Zhu, T. D. Raub, S. J. Daines, T. M. Lenton, Unique Neoproterozoic carbon isotope excursions sustained by coupled evaporite dissolution and pyrite burial. *Nat. Geosci.* **12**, 823–827 (2019).
52. T. A. Laakso, D. P. Schrag, A small marine biosphere in the Proterozoic. *Geobiology* **17**, 161–171 (2019).
53. T. M. Lenton, R. A. Boyle, S. W. Poulton, G. A. Shields-Zhou, N. J. Butterfield, Co-evolution of eukaryotes and ocean oxygenation in the Neoproterozoic era. *Nat. Geosci.* **7**, 257–265 (2014).
54. N. J. Butterfield, Oxygen, animals and oceanic ventilation: An alternative view. *Geobiology* **7**, 1–7 (2009).
55. C. Ma, Y. Tang, J. Ying, Global tectonics and oxygenation events drove the Earth-scale phosphorus cycle. *Earth Sci. Rev.* **233**, 104166 (2022).
56. F. Horton, Did phosphorus derived from the weathering of large igneous provinces fertilize the Neoproterozoic ocean? *Geochem. Geophys. Geosystems* **16**, 1723–1738 (2015).
57. L. J. Alcott, B. J. W. Mills, S. W. Poulton, Stepwise Earth oxygenation is an inherent property of global biogeochemical cycling. *Science* **366**, 1333–1337 (2019).
58. N. Flament, N. Coltice, P. F. Rey, The evolution of the 87Sr/86Sr of marine carbonates does not constrain continental growth. *Precambrian Res.* **229**, 177–188 (2013).
59. J. Korenaga, N. J. Planavsky, D. A. D. Evans, Global water cycle and the coevolution of the Earths interior and surface environment. *Philos. Trans. A. Math. Phys. Eng. Sci.* **375**, 20150393 (2017).
60. M. S. Dodd, Z. Zhang, C. Li, T. J. Algeo, T. W. Lyons, D. S. Hardisty, S. J. Loyd, D. L. Meyer, B. C. Gill, W. Shi, W. Wang, Development of carbonate-associated phosphate (CAP) as a proxy for reconstructing ancient ocean phosphate levels. *Geochim. Cosmochim. Acta* **301**, 48–69 (2021).
61. T. H. Green, E. B. Watson, Crystallization of apatite in natural magmas under high pressure, hydrous conditions, with particular reference to 'Orogenic' rock series. *Contrib. Mineral Petr.* **79**, 96–105 (1982).
62. J. Gaillardet, B. Dupré, P. Louvat, C. J. Allègre, Global silicate weathering and CO2 consumption rates deduced from the chemistry of large rivers. *Chem. Geol.* **159**, 3–30 (1999).
63. M. W. Bowler, M. J. Cliff, J. P. Waltho, G. M. Blackburn, Why did Nature select phosphate for its dominant roles in biology? *New J. Chem.* **34**, 784–794 (2010).
64. N. J. Planavsky, O. J. Rouxel, A. Bekker, S. V. Lalonde, K. O. Konhauser, C. T. Reinhard, T. W. Lyons, The evolution of the marine phosphate reservoir. *Nature* **467**, 1088–1090 (2010).
65. K. O. Konhauser, S. V. Lalonde, L. Amskold, H. D. Holland, Was there really an archean phosphate crisis? *Science* **315**, 1234 (2007).
66. W. Bleeker, The late Archean record: A puzzle in ca. 35 pieces. *Lithos* **71**, 99–134 (2003).
67. N. D. Greber, N. Dauphas, The chemistry of fine-grained terrigenous sediments reveals a chemically evolved Paleoarchean emerged crust. *Geochim. Cosmochim. Acta* **255**, 247–264 (2019).
68. S. M. McLennan, S. R. Taylor, Geochemical constraints on the growth of the continental crust. *J. Geol.* **90**, 347–361 (1982).
69. C. A. Partin, A. Bekker, N. J. Planavsky, C. T. Scott, B. C. Gill, C. Li, V. Podkovyrov, A. Maslov, K. O. Konhauser, S. V. Lalonde, G. D. Love, S. W. Poulton, T. W. Lyons, Large-scale

fluctuations in Precambrian atmospheric and oceanic oxygen levels from the record of U in shales. *Earth Planet. Sci. Lett.* **369-370**, 284–293 (2013).
70. C. T. Scott, A. Bekker, C. T. Reinhard, B. Schnetger, B. Krapež, D. Rumble III, T. W. Lyons, Late Archean euxinic conditions before the rise of atmospheric oxygen. *Geology* **39**, 119–122 (2011).
71. W. Lu, A. Ridgwell, E. Thomas, D. S. Hardisty, G. Luo, T. J. Algeo, M. R. Saltzman, B. C. Gill, Y. Shen, H.-F. Ling, C. T. Edwards, M. T. Whalen, X. Zhou, K. M. Gutchess, L. Jin, R. E. M. Rickaby, H. C. Jenkyns, T. W. Lyons, T. M. Lenton, L. R. Kump, Z. Lu, Late inception of a resiliently oxygenated upper ocean. *Science* **361**, 174–177 (2018).
72. C. W. Diamond, T. W. Lyons, Mid-Proterozoic redox evolution and the possibility of transient oxygenation events. *Emerg. Top Life Sci.* **2**, 235–245 (2018).
73. T. W. Lyons, C. T. Reinhard, N. J. Planavsky, The rise of oxygen in Earth's early ocean and atmosphere. *Nature* **506**, 307–315 (2014).
74. N. L. Swanson-Hysell, The Precambrian paleogeography of Laurentia. *Ancient Supercontinents Paleogeogr. Earth*, 109–153 (2021).

Acknowledgments: L.J., F., and S. Balon are thanked for their insightful comments on late drafts of the manuscript. E. Stueeken and R. Harrison are both thanked for keen insights that brought new clarity of vision to the manuscript. M. Kipp, K. Konhauser, and one anonymous reviewer are thanked for constructive and thoughtful reviews of the manuscript. **Funding:** C.R.W. acknowledges NERC and UKRI for support through a NERC DTP studentship grant number NE/L002507/1 and support in the form of a grant from the Leverhulme Centre for Life in the Universe. J.H. acknowledges National Key R&D Program of China (2021YFA0718200), CAS Hundred Talents Program, and CIFAR Azrieli Global Scholarship for the funding. **Author contributions:** Data acquisition, formal analysis, and data reduction: C.R.W., J.H., A.L., and S.E.P. Modeling, conceptualization, and figure production: C.R.W., J.H., O.S., and F.H. Manuscript drafting: C.R.W., J.H., F.E.J., A.L., F.H., H.W., A.L.Z., R.M.H., S.E.P., and O.S. Project supervision: O.S. and H.W. **Competing interests:** The authors declare that they have no competing interests. **Data and materials availability:** The Macrostrat database is freely available to query via the API, which contains instructions on data retrieval (https://macrostrat.org/api). The data underlying this work are permanently archived with the BGS NGDC (https://webapps.bgs.ac.uk/services/ngdc/accessions/index.html?_ga=2.179504036.1089183552.1622546055-1205285458.1610116506; deposit id F3F89F68074F719FE0530B37940A091A). All data needed to evaluate the conclusions in the paper are present in the paper and/or the Supplementary Materials.

Submitted 8 September 2022
Accepted 4 April 2023
Published 5 May 2023
10.1126/sciadv.ade6923
