# Supplementary material for: Evolution of the crustal phosphorus reservoir
Source: Sci Adv. 2023 May 5;9(18):eade6923. doi: 10.1126/sciadv.ade6923 (PMC10162663; doi:10.1126/sciadv.ade6923)
Supplement: Supplementary file 2 — Sections S1 to S3 Figs. S1 to S13 References [file sciadv.ade6923_sm.pdf]

Science Advances
AAAS

Supplementary Materials for

Evolution of the crustal phosphorus reservoir

Craig R. Walton *et al.*

Corresponding author: Craig R. Walton, crw59@cam.ac.uk; Jihua Hao, hao@ustc.edu.cn

Sci. Adv. **9**, eade6923 (2023)
DOI: 10.1126/sciadv.ade6923

This PDF file includes:

Sections S1 to S3
Figs. S1 to S13
References

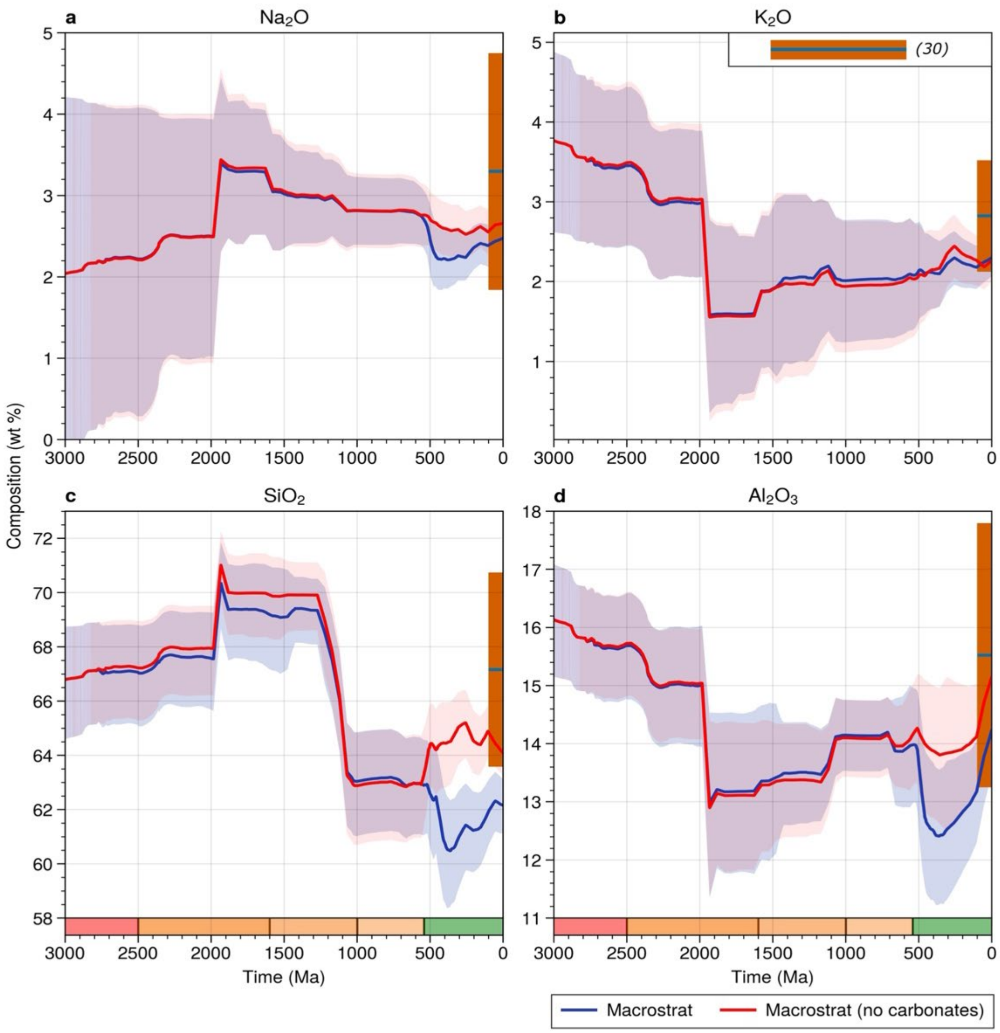

Fig. S1: **Reconstructed major element chemistry of w-CC over-time.** Uncertainties are 3 standard error. Rudnick and Gao (*30*) excluded carbonates from their analysis. Our estimates for Na_2O (a), K_2O (b), SiO_2 (c), and Al2O3 (d) are all within error of values of Rudnick and Gao (*30*)when carbonates are also excluded from our analysis.

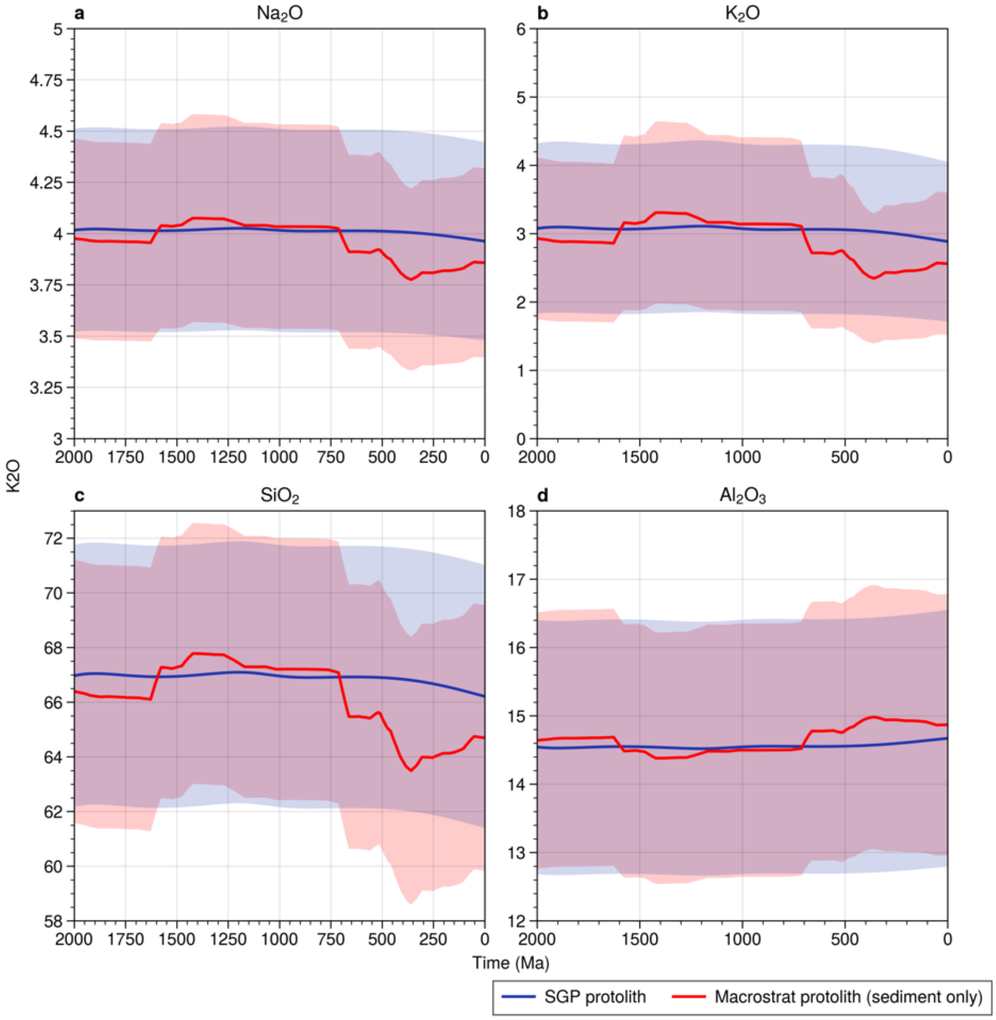

Fig. S2: **Major element comparison between Macrostrat sediments and the SGP database, after unweathering to obtain protolith compositions.** Uncertainties are 3 standard error for Macrostrat results and 1 sigma for SGP results. All results are within error.

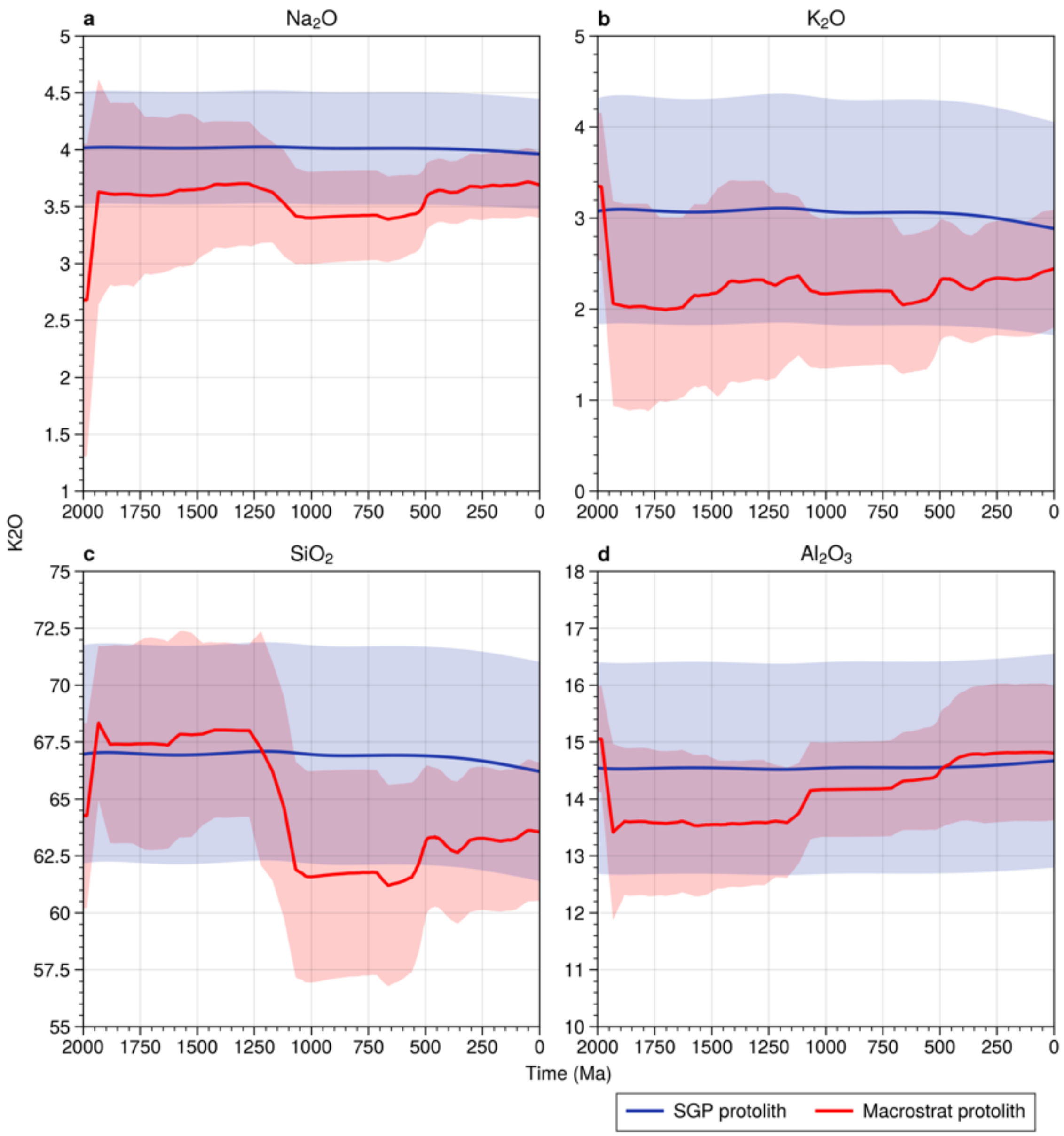

Fig.S3: **Major element comparison between Macrostrat (including igneous rocks) and the SGP database, after unweathering to obtain protolith compositions.** Uncertainties are 3 standard error for Macrostrat results and 1 sigma for SGP results. All results are within error.

S1. Design and caveats of global phosphorus weathering model

Life on Earth is at least in part limited by the oceanic availability of P (*40*, *63*). This may or may not have been true over much of Earth history (*32*, *64*, *65*). In order to test these scenarios, quantitative estimates of the crustal P weathering flux are needed. This flux is challenging to estimate due to uncertainties in how the key factors that determine it have changed over time. Several crucial factors are the solid Earth degassing flux, the P concentration within crustal rocks, and the weathering environment of those rocks (chemical and physical; Fig. 3).

Records of physical and chemical weathering environment over time are scarce, especially for the pre-Phanerozoic (*66*). Therefore, we do not present our results as a definitive estimate of the P weathering flux. For example, we lack reliable constraints on paleogeographic distribution of rock units, regional tectonic activity, and biological amplification of chemical weathering. Instead, we make a similar assumption to prior works (*1*, *3*, *67*); namely, that – over long timescales – average emergent crustal composition will be relevant for determining average P weathering fluxes. However, we note that our approach renders other aspects of P reservoir evolution tractable.

Here, we set out to understand the relevance of crustal lithochemical evolution in setting the global P weathering flux. One caveat for our approach is that weathering is not equally partitioned across Earth's continental surface area, as has been assumed in the simplified model. This places some important bounding constraints on how our results may be interpreted.

Unequal surface-partitioning of P weathering fluxes

Hartmann et al (*49*) show that 70% of P weathering flux comes from 10% of the total available land area. This evidence strongly supports the weighting of crustal composition (lithology-controlled weathering rates, P content) by the local tectonic and climatic factors that affect P transference into the oceans. Today, variable tectonics, climate, and soil cover together produce regional P chemical weathering hotspots (*49*). The modern P weathering flux in our analysis is sourced from around 70 % non-carbonate sediments, 20 % carbonates, and 10 % igneous rocks (Fig. 4), versus 41 % non-carbonate sediments, 14 % carbonates, and 45 % igneous rocks in Hartmann et al (*49*). This discrepancy owes both to our high P concentrations in post-Neoproterozoic sediments accounted for in our analysis, whilst Hartmann et al (*49*) employ global average rock unit compositions, as well as the absence of paleogeographic weathering hot spots in our model.

One take-away from this comparison is that bulk crust may not entirely reflect the composition of those rocks located in weathering hotspots at any given point in time. For example, young rocks may be more likely than ancient rocks to be contained within tectonically active weathering hotspots. However, in this case, P weathering would respond sensitively to changes in the composition of recently formed rocks. This would be a more extreme case than assumed in our work, which uses the bulk crustal composition to model P weathering fluxes. If young rocks are very important as P sources at any given point in time, then the P cycle will have been modulated by lithochemical evolution even more rapidly and dramatically than we modelled. This outcome would represent strong support for the hypothesis that we set out to test.

The approach of Hartmann et al (*49*) considers lithology specific weathering rates and runoff variations (i.e., climate) that we do not account for. This is due to the great difficulty of reliably estimating such factors across deep time. However, crustal lithochemistry is nonetheless an important factor in the global P cycle. Provided that the P content of average emergent crust is

not hugely different from that of the crust contained in weathering hotspots, a similar value to the actual global P weathering flux should be obtained in a simplified model considering only total CO_2 consumed during weathering per year. This fact underpins our approach and explains why we are able to recover within error the modern P weathering flux without considering all factors that certainly are relevant for a detailed description of the global P cycle today. Our weathering model would heavily underestimate the observed modern P weathering flux without the strong contribution by relatively P-rich post-Neoproterozoic sediments. Therefore, sediment accumulation and compositional evolution emerge as important factors in shaping the P cycle over long timescales.

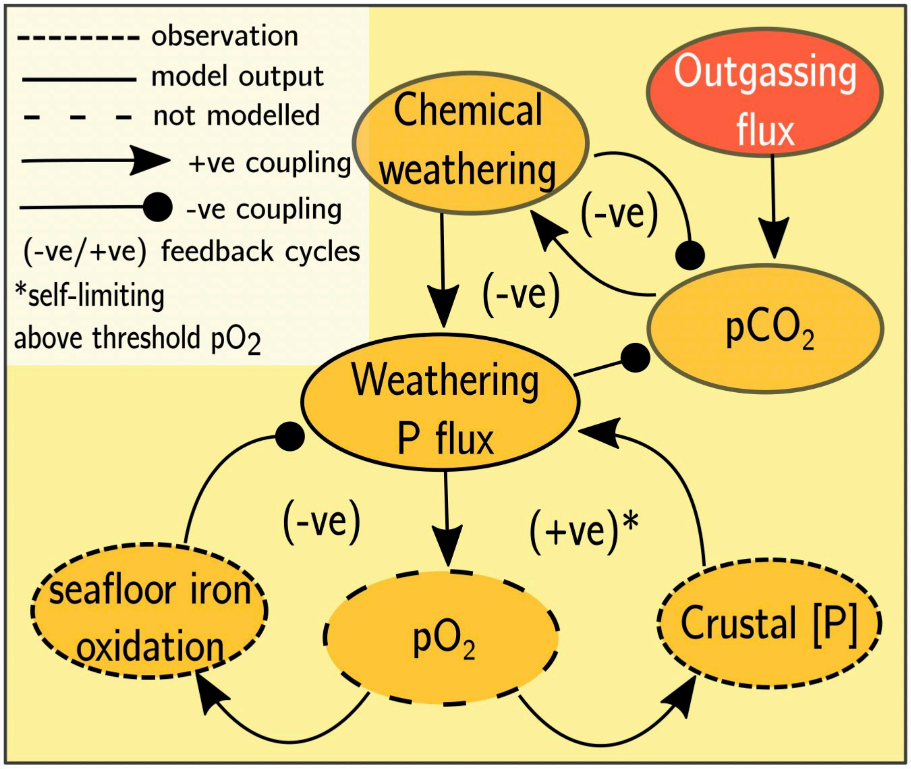

Fig. S4: **Important feedbacks capable of modifying the P cycle after the origin of oxygenic photosynthesis.** Negative feedbacks operate that prevent sustained increase in P weathering via higher pCO_2 without a matching permanent increase in outgassing flux. The fraction of total P supply used to fuel oxygenic photosynthesis drives a second negative (-ve) feedback: enhancing seafloor iron oxidation and driving hydrothermal P burial. Finally, a positive (+ve) feedback (within some redox regime) may operate between pO_2 and global P weathering flux via the reweathering of marginal sediments enriched in biologically recycled P.

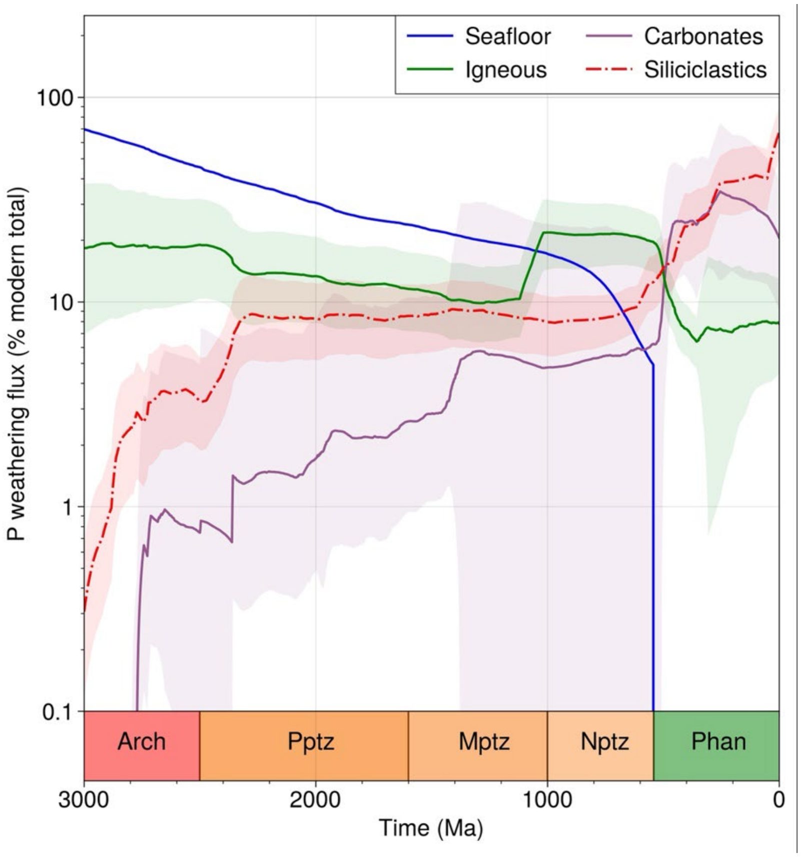

Fig. S5: **output of weathering model with rapid land emergence from Korenaga et al (*59*) and (*68*).** Structure of the record is nearly identical to that obtained using Flament (*50*) (see main text). Precambrian P fluxes are similar, but do not drop off as precipitously in the Archean in the land-only model.

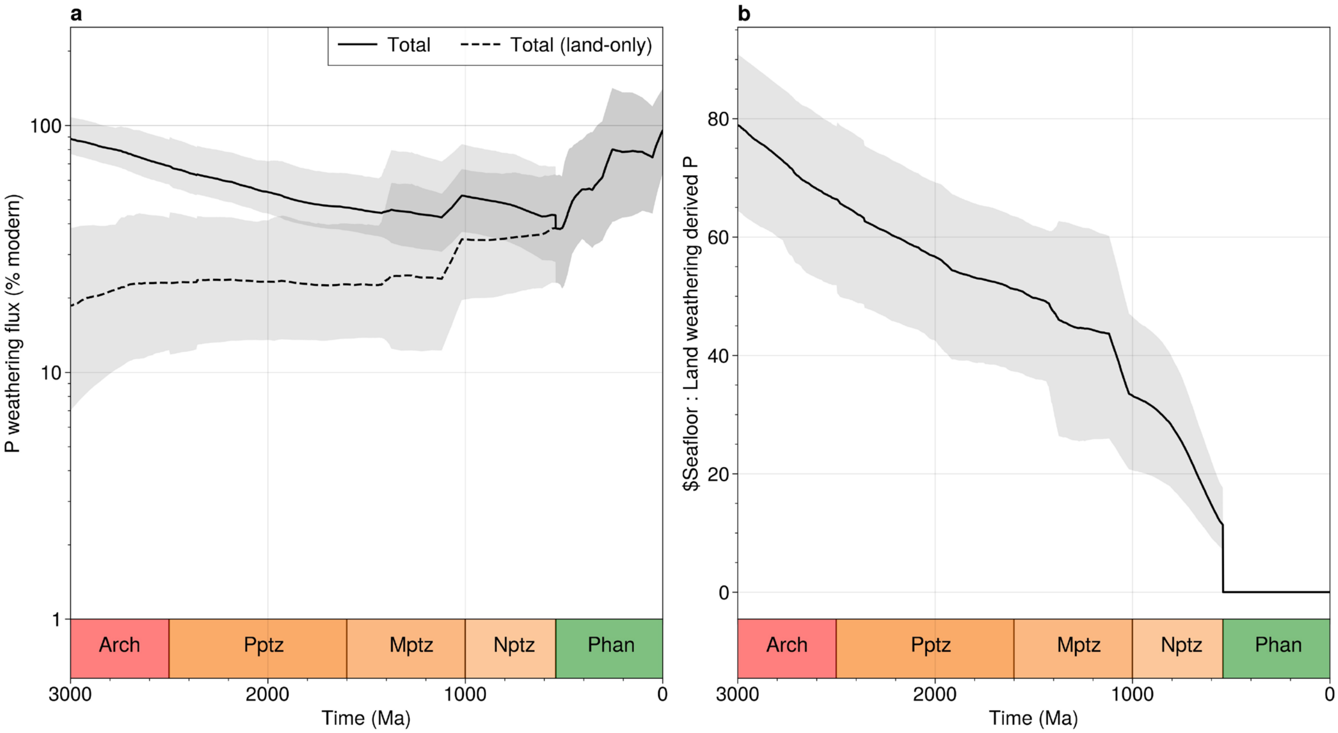

Fig. S6: **output of weathering model with rapid land emergence from Korenaga et al (*59*) and (*68*).** Structure of the record is nearly identical to that obtained using Flament (*50*) (see main text). Precambrian P fluxes are similar, but do not drop off as precipitously in the Archean in the land-only model.

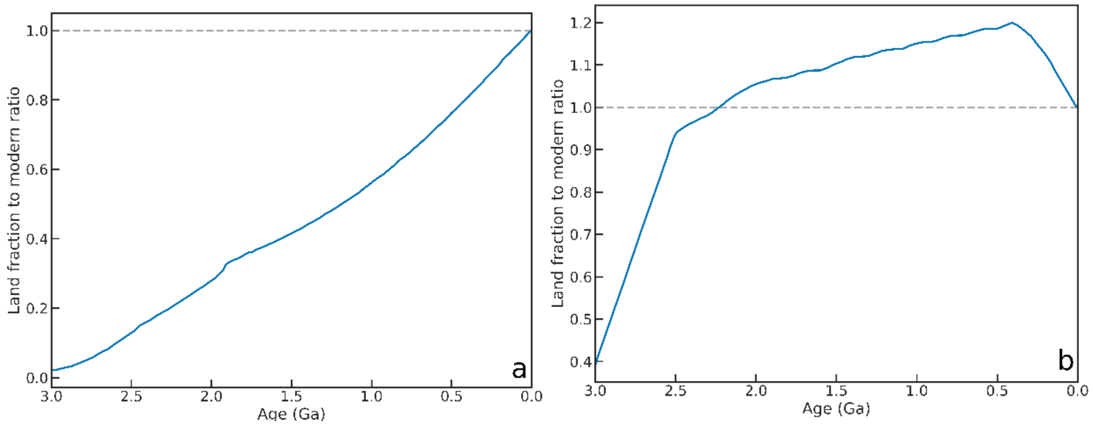

Figure S7. **Land emergence history as input of the weathering model:** a. slow emergence of continental land reconstructed by Flament et al (*50*); b. rapid emergence of continental land reconstructed by Korenaga et al. (*59*) using the crustal evolution history by McLennan & Taylor (*68*).

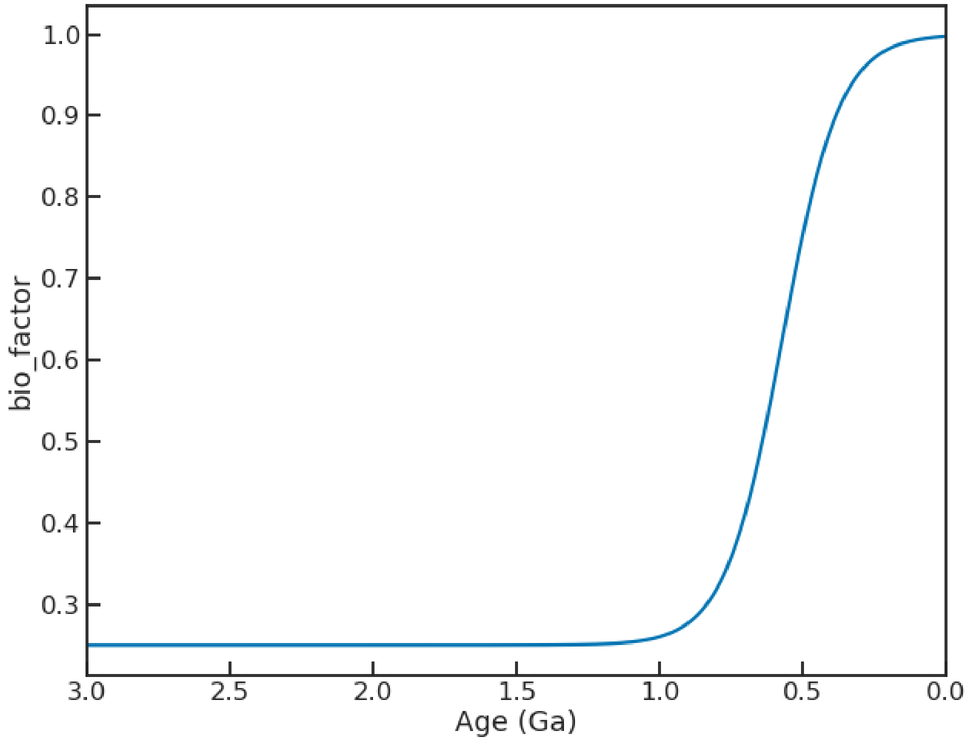

Figure S8. **Biological compensating factor used in this model.**

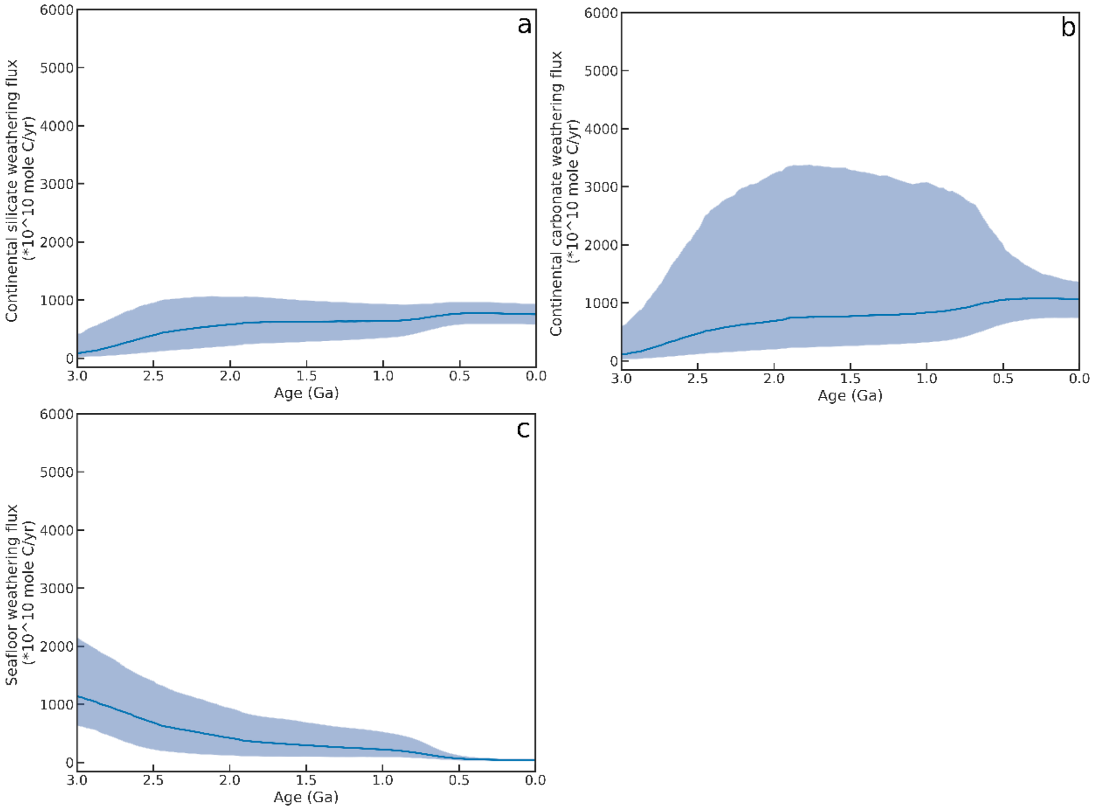

Fig. S9. **Weathering flux outputs by assuming the slow emergence of continental land** (*50*).

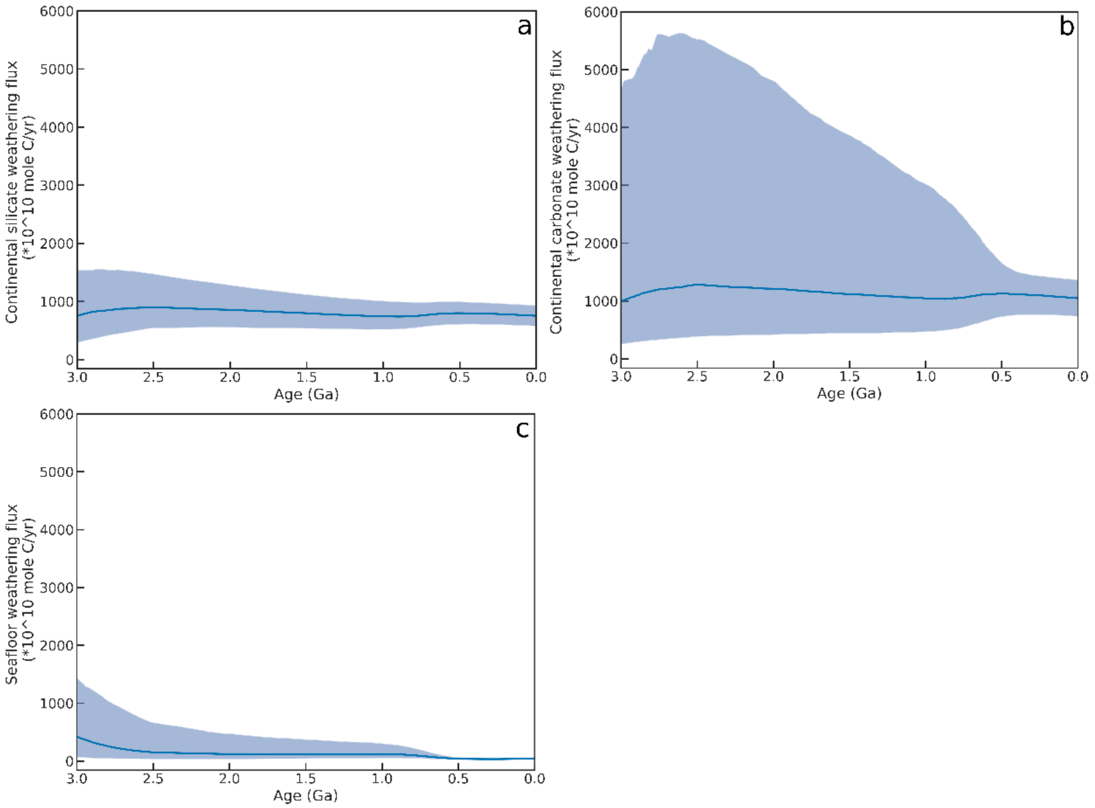

Fig. S10. **Weathering flux outputs by assuming the rapid emergence of continental land** (*59*).

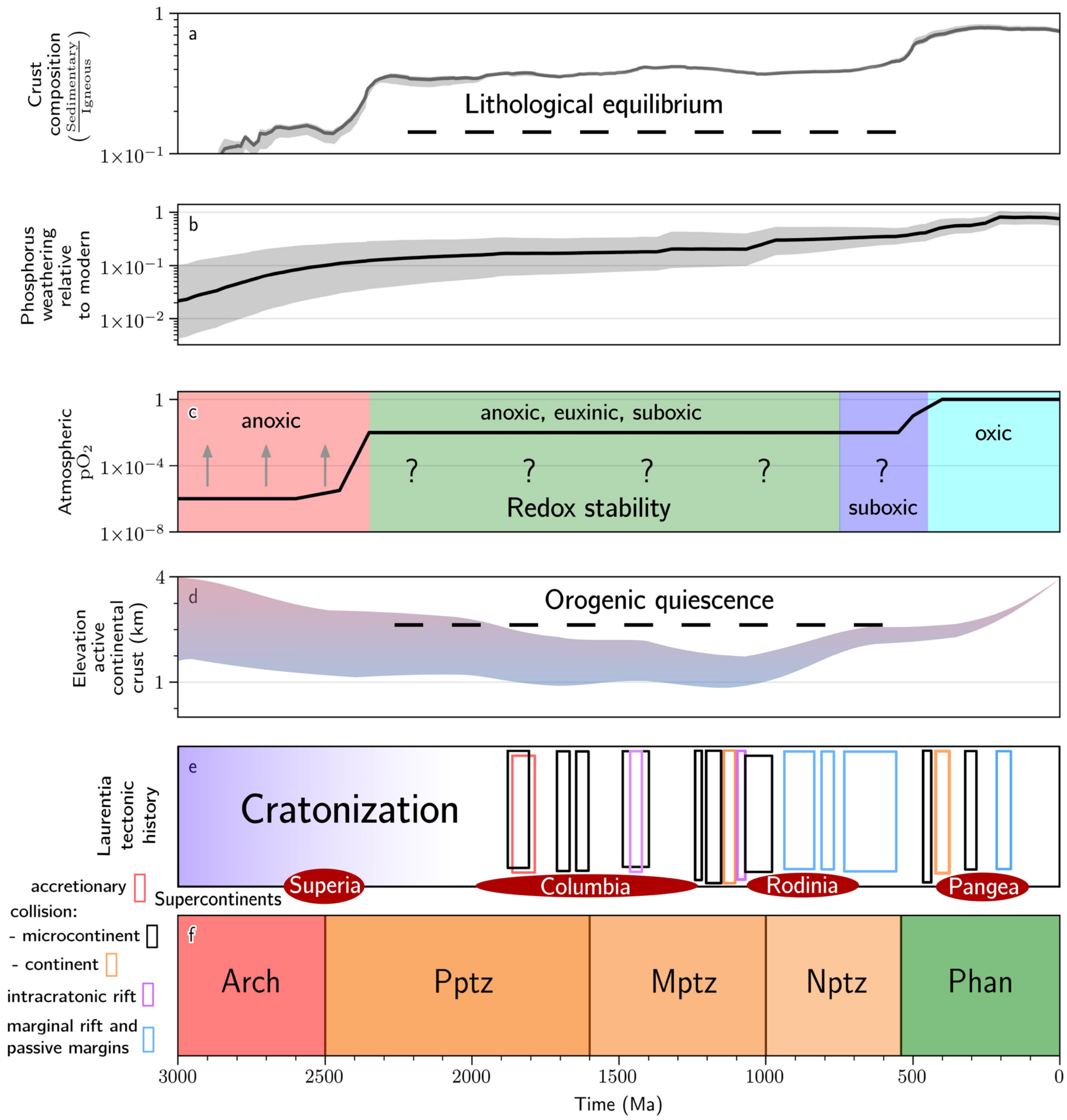

Fig. S11: **Context of sediment accumulation and P weathering within long term Earth surface evolution.** a) Accumulation of sedimentary relative to igneous rock within weatherable continental crust, as reconstructed in this work. Two major episodes of accumulation occur, at the Archean-Proterozoic and Neoproterozoic-Phanerozoic boundary. b) Steady increase in P weathering supply to the biosphere estimated in this work (land weathering only), with long periods of stagnation, and a relatively rapid increase to modern values across the early Phanerozoic. c) Redox evolution of Earth surface environments (*48*). Shaded and labelled regions describe the redox evolution of the oceans (*32*, *38*, *69*–*73*). d) Orogenic activity over time, as reconstructed by Tang et al (*21*). e) North American (Laurentian) tectonic evolution, showing early sustained cratonization, followed by a more detailed history of extensional and compressional regimes, and at a smaller scale geographically disparate and discrete tectonic events, underlined with estimated temporal limits of supercontinents (*74*). f) Coarse-scale geological timeline of Earth history

S2. Scenarios for interpreting the Macrostrat record of lithological proportions

The Macrostrat record of lithological proportions (Fig. 1, main text) is a valuable constraint on the balance of rock accumulation versus destruction over time. However, without prior knowledge of the original proportions of rock types, it is difficult to quantitatively interpret the record with certainty.

In Figure S12a-f, we illustrate three (schematic) scenarios where structure in f_{sed} is determined largely by bias induced in two major erosive events (a-c). We do not illustrate any scenarios where background weathering induces bias progressively with age, as this appears inconsistent with our findings. In these bias-driven scenarios, sediment production may have been steady, have changed, or have been strongly linked to destruction, i.e., extensive preservation of reworked material. In each case, the stepped structure that we observe using Macrostrat (Fig. 1a) is potentially possible to reproduce. Structure in this case derives completely or mostly from bias, and a fraction of the reconstructed change in crustal P concentration would be an artefact: partially, but necessarily not completely, being owed to apparent differences in f_{sed} over time.

We also illustrate scenarios where the majority of change in f_{sed} derives from real differences over time in production (d-f). The largest changes are possible where changes in production occur coupled to and during as well as permanently following major erosive episodes (f). The shape of the f_{sed} record can be expected to change depending on whether changes in f_{sed} of recently formed crust are transient (d) or permanent (e-f).

Specifically considering the relevance of the crustal P enrichment mechanisms discussed in our work, scenario a) is least favorable. In this case, all of the apparent variation in f_{sed} over time is a preservation bias, meaning that the contribution by sediments to the crustal P reservoir will be heavily underestimated for the Precambrian. This would translate to a lower bound scenario for increasing crustal P relative to early times entering the Phanerozoic. A difference would still occur, due to the falling contribution of ancient crust and changing P content of young sediments, and our estimate of P weathering – lacking any correction of ancient crustal segments for erosional biases – would become increasingly accurate moving towards the present (where what is preserved now is necessarily more representative of what was available for weathering, then).

Considering the size of the changes in f_{sed} in macrostrat, this might translate to underestimating the Archean weathering contribution by sediments by 5-to-10-fold. However, given that sediments and igneous rocks do not differ in P concentration hugely in the Archean, and that our model is not sensitive to the chemical weathering rates of siliciclastic versus igneous lithologies, consequently higher igneous P weathering fluxes would largely balance out the lower sedimentary weathering P flux. Indeed, the major change across the Neoproterozoic-Phanerozoic boundary would still manifest, given the loss of old low P sediment (in this case rapidly relative to old igneous rocks). So, even though the mechanics of the scenario differ from the one we favor, the outcome for P cycling would be quite similar. The main difference would be for the role of carbonates, which play a regulatory role in the overall C cycle – underestimation of which would be an error in the weathering model, with probable knock-on effects on P weathering, and demanding some form of correction.

Scenario 1f is the most favorable for our proposed crustal P-enriching mechanism. 1f is extreme in that old P-poor material is lost, fresh material (potentially P-rich) is created from it, and long-term step-changes in sediment production occur. All of the change in f_{sed} is real, with no

bias with respect to lithology being imparted. This scenario, which we favour, provides the least demanding physical constraints on reworking the crustal P reservoir, i.e., using a combination of processes to engender changes in crustal P.

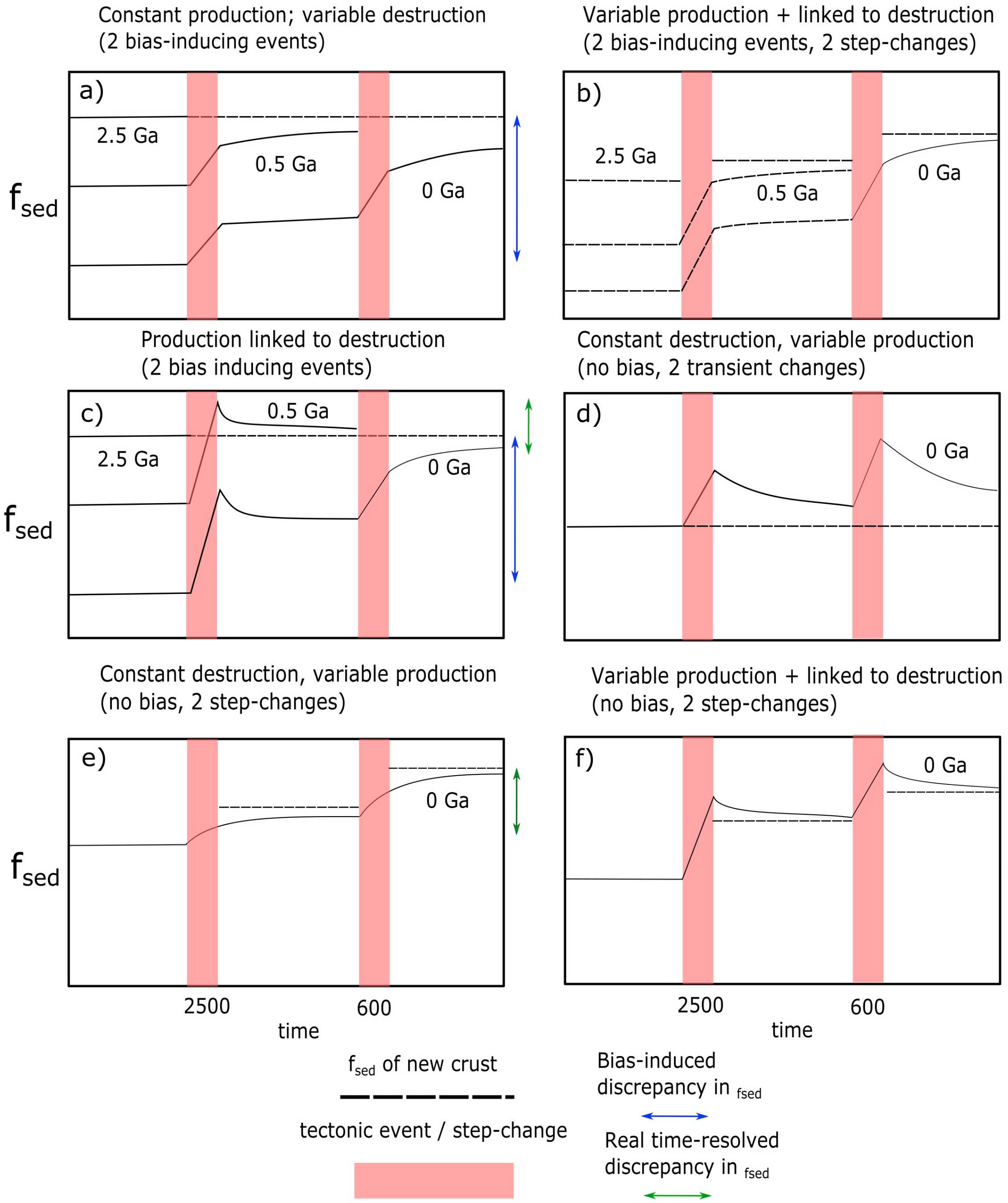

Fig. S12: Schematic scenarios for f_{sed} fraction of the rock record. Variable destruction is used to mean that lithology-specific erosion rates vary independently during certain tectonic events. Production linked to destruction indicates that focussed episodes of bias-inducing erosion may produce a large volume of sediment derived from the eroded older crust, potentially producing an 'overshoot' in fsed which is then gradually eliminated as a steady state destruction to production ratio of sedimentary/igneous rocks is tended toward.

S3. Lag-time between sediment deposition and exhumation

We have avoided overly specific causal interpretations of our results, focussing on what likely cannot be explained given the timing of P-enrichment (see Discussion). However, it is clear that the sustained increase in crustal P concentration may be of relevance to various biogeochemical changes in the Phanerozoic. Determining cause and effect in this context will require caution when interpreting the rock record. As an important example, our work translates crustal composition instantaneously into estimated P fluxes, i.e., there is no delay between the formation of a rock unit and its potential contribution to weathering in the model as formulated. However, for most lithologies there is some necessary lag-time between formation and active contribution to weathering. Hence, changes in crustal nutrient concentration may only impact the biosphere after some tens of millions of years.

Weathering lag-times of tens-to-hundreds of Myr would result in a geologically meaningful delay between biologically- and erosively-driven P-enrichment of the crust and increased global P weathering fluxes. Considering nuanced factors of rock record interpretation such as the above may therefore be crucial when attempting to explore and quantify links between changes in the geosphere and the biosphere. This point is especially important for the Phanerozoic, when independent events of biogeochemical changes occur within ten to one hundred Myr of one another.

We quantify the possible importance of exhumation lag times by randomly adding one sided error to actual unit ages. This is done by randomly adding one-sided error pulled from a uniform distribution (three separate scenarios: 0-10, 0-100, and 0-1000 Myr lag). This process was repeated 100 times and the mean trajectories for P concentration in weatherable crust calculated. Results are shown in Fig. S12. Associated 2 standard deviation error bars are shown as blue shaded regions.

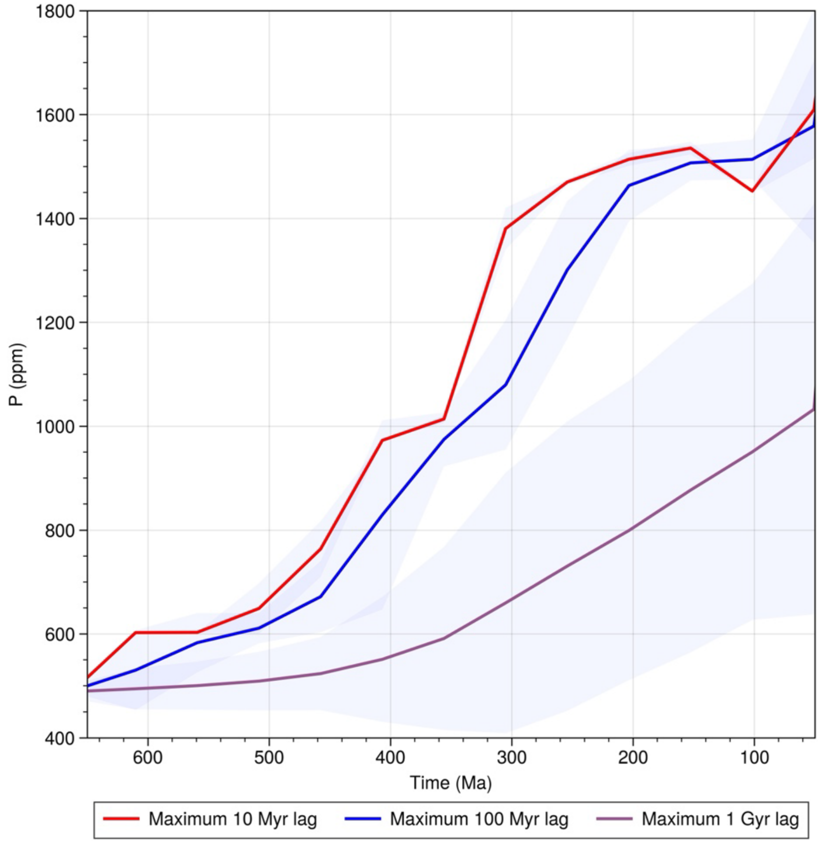

Fig. S13: Effect of lag-times between sedimentary unit formation and participation in weathering on the P concentration of weatherable continental crust.

These sensitivity tests show that the shift in crustal P identified in our work will still occur but would be delayed – and hence also delayed would be the higher baseline P weathering flux. This outcome is relevant in pursuit of the actual history of P weathering and hence attempts to e.g., explain the timeline of atmospheric pO_2. Similarly, this result adds weight to our argument that enhanced P weathering due to increasingly P-rich weatherable crust is not a viable cause-effect relationship to trigger earlier oxygenation events. That being said, the possible delay would shift the increase in P supply closer in line with later Phanerozoic step-changes in pO2 reconstructed with proxy records and box models, e.g., Tostevin & Mills *(48)*.

REFERENCES

1. G. M. Cox, T. W. Lyons, R. N. Mitchell, D. Hasterok, M. Gard, Linking the rise of atmospheric oxygen to growth in the continental phosphorus inventory. *Earth Planet. Sci. Lett.* **489**, 28–36 (2018).

2. K. J. Zahnle, D. C. Catling, M. W. Claire, The rise of oxygen and the hydrogen hourglass. *Chem. Geol.* **362**, 26–34 (2013).

3. N. D. Greber, N. Dauphas, A. Bekker, M. P. Ptáček, I. N. Bindeman, A. Hofmann, Titanium isotopic evidence for felsic crust and plate tectonics 3.5 billion years ago. *Science* **357**, 1271–1274 (2017).

4. A. G. Lipp, O. Shorttle, E. A. Sperling, J. J. Brocks, D. B. Cole, P. W. Crockford, L. D. Mouro, K. Dewing, S. Q. Dornbos, J. F. Emmings, U. C. Farrell, A. Jarrett, B. W. Johnson, P. Kabanov, C. B. Keller, M. Kunzmann, A. J. Miller, N. T. Mills, B. O'Connell, S. E. Peters, N. J. Planavsky, S. R. Ritzer, S. D. Schoepfer, P. R. Wilby, J. Yang, The composition and weathering of the continents over geologic time. *Geochem. Perspect. Lett.* **7**, 21–26 (2021).

5. K. C. Condie, Chemical composition and evolution of the upper continental crust: Contrasting results from surface samples and shales. *Chem. Geol.* **104**, 1–37 (1993).

6. G. J. S. Bluth, L. R. Kump, Phanerozoic paleogeology. *Am. J. Sci.* **291**, 284–308 (1991).

7. S. E. Peters, C. R. Walton, J. M. Husson, D. P. Quinn, O. Shorttle, C. B. Keller, R. R. Gaines, Igneous rock area and age in continental crust. *Geology* **49**, 1235–1239 (2021).

8. E. E. Stüeken, R. Buick, R. E. Anderson, J. A. Baross, N. J. Planavsky, T. W. Lyons, Environmental niches and metabolic diversity in Neoarchean lakes. *Geobiology* **15**, 767–783 (2017).

9. J. M. Husson, S. E. Peters, Atmospheric oxygenation driven by unsteady growth of the continental sedimentary reservoir. *Earth Planet. Sci. Lett.* **460**, 68–75 (2017).

10. S. E. Peters, J. M. Husson, J. Czaplewski, Macrostrat: A platform for geological data integration and deep-time earth crust research. *Geochem. Geophys. Geosystems* **19**, 1393–1409 (2018).

11. J. M. Husson, S. E. Peters, Nature of the sedimentary rock record and its implications for Earth system evolution. *Emerg. Top Life Sci.* **2**, 125–136 (2018).

12. S. E. Peters, J. M. Husson, Sediment cycling on continental and oceanic crust. *Geology* **45**, 323–326 (2017).

13. C. P. Slomp, P. V. Cappellen, The global marine phosphorus cycle: Sensitivity to oceanic circulation. *Biogeosciences* **4**, 10.5194/bgd-3-1587-2006 (2007).

14. T. A. Laakso, E. A. Sperling, D. T. Johnston, A. H. Knoll, Ediacaran reorganization of the marine phosphorus cycle. *Proc. Natl. Acad. Sci. U.S.A.* **117**, 11961–11967 (2020).

15. N. Moosdorf, S. Cohen, C. von Hagke, A global erodibility index to represent sediment production potential of different rock types. *Appl. Geogr.* **101**, 36–44 (2018).

16. B. Campforts, V. Vanacker, F. Herman, M. Vanmaercke, W. Schwanghart, G. E. Tenorio, P. Willems, G. Govers, Parameterization of river incision models requires accounting for environmental heterogeneity: Insights from the tropical Andes. *Earth Surf. Dynam.* **8**, 447–470 (2020).

17. A. M. Mloszewska, S. J. Mojzsis, E. Pecoits, D. Papineau, N. Dauphas, K. O. Konhauser, Chemical sedimentary protoliths in the >3.75Ga Nuvvuagittuq Supracrustal Belt (Québec, Canada). *Gondw. Res.* **23**, 574–594 (2013).

18. J. E. J. Martini, A late Archaean-Palaeoproterozoic (2.6 Ga) palaeosol on ultramafics in the Eastern Transvaal, South Africa. *Precambrian Res.* **67**, 159–180 (1994).

19. B. Ausín, E. Bruni, N. Haghipour, C. Welte, S. M. Bernasconi, T. I. Eglinton, Controls on the abundance, provenance and age of organic carbon buried in continental margin sediments. *Earth Planet. Sci. Lett.* **558**, 116759 (2021).

20. J. F. Read, Carbonate platforms of passive (extensional) continental margins: Types, characteristics and evolution. *Tectonophysics* **81**, 195–212 (1982).

21. M. Tang, X. Chu, J. Hao, B. Shen, Orogenic quiescence in Earth's middle age. *Science* **371**, 728–731 (2021).

22. P. Chowdhury, J. A. Mulder, P. A. Cawood, S. Bhattacharjee, S. Roy, A. N. Wainwright, O. Nebel, S. Mukherjee, Magmatic thickening of crust in non–plate tectonic settings initiated the subaerial rise of Earth's first continents 3.3 to 3.2 billion years ago. *Proc. Natl. Acad. Sci. U.S.A.* **118**, e2105746118 (2021).

23. S. E. Peters, R. R. Gaines, Formation of the 'Great Unconformity' as a trigger for the Cambrian explosion. *Nature* **484**, 363–366 (2012).

24. C. B. Keller, J. M. Husson, R. N. Mitchell, W. F. Bottke, T. M. Gernon, P. Boehnke, E. A. Bell, N. L. Swanson-Hysell, S. E. Peters, Neoproterozoic glacial origin of the Great Unconformity. *Proc. Natl. Acad. Sci. U.S.A.* **116**, 1136–1145 (2018).

25. K. T. McDannell, C. B. Keller, W. R. Guenthner, P. K. Zeitler, D. L. Shuster, Thermochronologic constraints on the origin of the Great Unconformity. *Proc. Natl. Acad. Sci. U.S.A.* **119**, e2118682119 (2022).

26. A. B. Ronov, V. E. Khain, A. N. Balukhovsky, K. B. Seslavinsky, Quantitative analysis of Phanerozoic sedimentation. *Sediment. Geol.* **25**, 311–325 (1980).

27. G. G. Roberts, N. White, B. H. Lodhia, The generation and scaling of longitudinal river profiles. *J Geophys. Res Earth Surf.* **124**, 137–153 (2019).

28. J. Veizer, F. T. Mackenzie, Treatise on Geochemistry, 369–407 (2003).

29. M. Tang, K. Chen, R. L. Rudnick, Archean upper crust transition from mafic to felsic marks the onset of plate tectonics. *Science* **351**, 372–375 (2016).

30. R. L. Rudnick, S. Gao, Composition of the Continental *Crust. Geochemistry* **3**, 659 (2014).

31. A. G. Lipp, O. Shorttle, F. Syvret, G. G. Roberts, Major element composition of sediments in terms of weathering and provenance: Implications for crustal recycling. *Geochem. Geophys. Geosystems* **21**, e2019GC008758 (2020).

32. C. T. Reinhard, N. J. Planavsky, B. C. Gill, K. Ozaki, L. J. Robbins, T. W. Lyons, W. W. Fischer, C. Wang, D. B. Cole, K. O. Konhauser, Evolution of the global phosphorus cycle. *Nature* **541**, 386–389 (2017).

33. N. J. Planavsky, D. Asael, A. D. Rooney, L. J. Robbins, B. C. Gill, C. M. Dehler, D. B. Cole, S. M. Porter, G. D. Love, K. O. Konhauser, C. T. Reinhard, A sedimentary record of the evolution of the global marine phosphorus cycle. *Geobiology* **21**, 168–174 (2023).

34. K. O. Konhauser, E. Pecoits, S. V. Lalonde, D. Papineau, E. G. Nisbet, M. E. Barley, N. T. Arndt, K. Zahnle, B. S. Kamber, Oceanic nickel depletion and a methanogen famine before the Great Oxidation Event. *Nature* **458**, 750–753 (2009).

35. K. O. Konhauser, L. J. Robbins, E. Pecoits, C. Peacock, A. Kappler, S. V. Lalonde, The Archean nickel famine revisited. *Astrobiology* **15**, 804–815 (2015).

36. E. E. Stüeken, D. C. Catling, R. Buick, Contributions to late Archaean sulphur cycling by life on land. *Nat. Geosci.* **5**, 722–725 (2012).

37. N. J. Planavsky, D. Asael, A. Hofmann, C. T. Reinhard, S. V. Lalonde, A. Knudsen, X. Wang, F. O. Ossa, E. Pecoits, A. J. B. Smith, N. J. Beukes, A. Bekker, T. M. Johnson, K. O. Konhauser, T. W. Lyons, O. J. Rouxel, Evidence for oxygenic photosynthesis half a billion years before the Great Oxidation Event. *Nat. Geosci.* **7**, 283–286 (2014).

38. A. C. Johnson, C. M. Ostrander, S. J. Romaniello, C. T. Reinhard, A. T. Greaney, T. W. Lyons, A. D. Anbar, Reconciling evidence of oxidative weathering and atmospheric anoxia on Archean Earth. *Sci. Adv.* **7**, eabj0108 (2021).

39. M. Thoby, K. O. Konhauser, P. W. Fralick, W. Altermann, P. T. Visscher, S. V. Lalonde, Global importance of oxic molybdenum sinks prior to 2.6 Ga revealed by the Mo isotope composition of Precambrian carbonates. *Geology* **47**, 559–562 (2019).

40. A. Paytan, K. McLaughlin, The oceanic phosphorus cycle. *Chem. Rev.* **107**, 563–576 (2007).

41. J. S. Compton, E. W. Bergh, Phosphorite deposits on the Namibian shelf. *Mar. Geol.* **380**, 290–314 (2016).

42. M. A. Kipp, E. E. Stüeken, Biomass recycling and Earth's early phosphorus cycle. *Sci. Adv.* **3**, eaao4795 (2017).

43. J. Hao, A. H. Knoll, F. Huang, R. M. Hazen, I. Daniel, Cycling phosphorus on the archean earth: Part I. Continental weathering and riverine transport of phosphorus. *Geochim. Cosmochim. Acta* **273**, 70–84 (2020).

44. J. Krissansen-Totton, G. N. Arney, D. C. Catling, Constraining the climate and ocean pH of the early Earth with a geological carbon cycle model. *Proc. Natl. Acad. Sci. U.S.A.* **115**, 4105–4110 (2018).

45. D. D. Syverson, C. T. Reinhard, T. T. Isson, C. J. Holstege, J. A. R. Katchinoff, B. M. Tutolo, B. Etschmann, J. Brugger, N. J. Planavsky, Nutrient supply to planetary biospheres from anoxic weathering of mafic oceanic crust. *Geophys. Res. Lett.* **48** e2021GL094442 (2021).

46. C. G. Wheat, J. McManus, M. J. Mottl, E. Giambalvo, Oceanic phosphorus imbalance: Magnitude of the mid-ocean ridge flank hydrothermal sink. *Geophys. Res. Lett.* **30**, 10.1029/2003GL017318 (2003).

47. C. G. Wheat, R. A. Feely, M. J. Mottl, Phosphate removal by oceanic hydrothermal processes: An update of the phosphorus budget in the oceans. *Geochim. Cosmochim. Acta* **60**, 3593–3608 (1996).

48. R. Tostevin, B. J. W. Mills, Reconciling proxy records and models of Earth's oxygenation during the Neoproterozoic and Palaeozoic. *Interface Focus* **10**, 20190137 (2020).

49. J. Hartmann, N. Moosdorf, R. Lauerwald, M. Hinderer, A. J. West, Global chemical weathering and associated P-release — The role of lithology, temperature and soil properties. *Chem. Geol.* **363**, 145–163 (2014).

50. J. J. Williams, B. J. W. Mills, T. M. Lenton, A tectonically driven Ediacaran oxygenation event. *Nat. Commun.* **10**, 2690 (2019).

51. G. A. Shields, B. J. W. Mills, M. Zhu, T. D. Raub, S. J. Daines, T. M. Lenton, Unique Neoproterozoic carbon isotope excursions sustained by coupled evaporite dissolution and pyrite burial. *Nat. Geosci.*, **12**, 823–827 (2019).

52. T. A. Laakso, D. P. Schrag, A small marine biosphere in the Proterozoic. *Geobiology* **17**, 161–171 (2019).

53. T. M. Lenton, R. A. Boyle, S. W. Poulton, G. A. Shields-Zhou, N. J. Butterfield, Co-evolution of eukaryotes and ocean oxygenation in the Neoproterozoic era. *Nat. Geosci.* **7**, 257–265 (2014).

54. N. J. Butterfield, Oxygen, animals and oceanic ventilation: An alternative view. *Geobiology* **7**, 1–7 (2009).

55. C. Ma, Y. Tang, J. Ying, Global tectonics and oxygenation events drove the Earth-scale phosphorus cycle. *Earth Sci. Rev.* **233**, 104166 (2022).

56. F. Horton, Did phosphorus derived from the weathering of large igneous provinces fertilize the Neoproterozoic ocean? *Geochem. Geophys. Geosystems* **16**, 1723–1738 (2015).

57. L. J. Alcott, B. J. W. Mills, S. W. Poulton, Stepwise Earth oxygenation is an inherent property of global biogeochemical cycling. *Science* **366**, 1333–1337 (2019).

58. N. Flament, N. Coltice, P. F. Rey, The evolution of the 87Sr/86Sr of marine carbonates does not constrain continental growth. *Precambrian Res.* **229**, 177–188 (2013).

59. J. Korenaga, N. J. Planavsky, D. A. D. Evans, Global water cycle and the coevolution of the Earths interior and surface environment. *Philos. Trans. A. Math. Phys. Eng. Sci.* **375**, 20150393 (2017).

60. M. S. Dodd, Z. Zhang, C. Li, T. J. Algeo, T. W. Lyons, D. S. Hardisty, S. J. Loyd, D. L. Meyer, B. C. Gill, W. Shi, W. Wang, Development of carbonate-associated phosphate (CAP) as a proxy

for reconstructing ancient ocean phosphate levels. *Geochim. Cosmochim. Acta* **301**, 48–69 (2021).

61. T. H. Green, E. B. Watson, Crystallization of apatite in natural magmas under high pressure, hydrous conditions, with particular reference to 'Orogenic' rock series. *Contrib. Mineral Petr.* **79**, 96–105 (1982).

62. J. Gaillardet, B. Dupré, P. Louvat, C. J. Allègre, Global silicate weathering and CO2 consumption rates deduced from the chemistry of large rivers. *Chem. Geol.* **159**, 3–30 (1999).

63. M. W. Bowler, M. J. Cliff, J. P. Waltho, G. M. Blackburn, Why did Nature select phosphate for its dominant roles in biology? *New J. Chem.* **34**, 784–794 (2010).

64. N. J. Planavsky, O. J. Rouxel, A. Bekker, S. V. Lalonde, K. O. Konhauser, C. T. Reinhard, T. W. Lyons, The evolution of the marine phosphate reservoir. *Nature* **467**, 1088–1090 (2010).

65. K. O. Konhauser, S. V. Lalonde, L. Amskold, H. D. Holland, Was there really an archean phosphate crisis? *Science* **315**, 1234 (2007).

66. W. Bleeker, The late Archean record: A puzzle in ca. 35 pieces. *Lithos* **71**, 99–134 (2003).

67. N. D. Greber, N. Dauphas, The chemistry of fine-grained terrigenous sediments reveals a chemically evolved Paleoarchean emerged crust. *Geochim. Cosmochim. Acta* **255**, 247–264 (2019).

68. S. M. McLennan, S. R. Taylor, Geochemical constraints on the growth of the continental crust. *J. Geol.* **90**, 347–361 (1982).

69. C. A. Partin, A. Bekker, N. J. Planavsky, C. T. Scott, B. C. Gill, C. Li, V. Podkovyrov, A. Maslov, K. O. Konhauser, S. V. Lalonde, G. D. Love, S. W. Poulton, T. W. Lyons, Large-scale fluctuations in Precambrian atmospheric and oceanic oxygen levels from the record of U in shales. *Earth Planet. Sci. Lett.* **369-370**, 284–293 (2013).

70. C. T. Scott, A. Bekker, C. T. Reinhard, B. Schnetger, B. Krapež, D. Rumble III, T. W. Lyons, Late Archean euxinic conditions before the rise of atmospheric oxygen. *Geology* **39**, 119–122 (2011).

71. W. Lu, A. Ridgwell, E. Thomas, D. S. Hardisty, G. Luo, T. J. Algeo, M. R. Saltzman, B. C. Gill, Y. Shen, H.-F. Ling, C. T. Edwards, M. T. Whalen, X. Zhou, K. M. Gutchess, L. Jin, R. E. M. Rickaby, H. C. Jenkyns, T. W. Lyons, T. M. Lenton, L. R. Kump, Z. Lu, Late inception of a resiliently oxygenated upper ocean. *Science* **361**, 174–177 (2018).

72. C. W. Diamond, T. W. Lyons, Mid-Proterozoic redox evolution and the possibility of transient oxygenation events. *Emerg. Top Life Sci.* **2**, 235–245 (2018).

73. T. W. Lyons, C. T. Reinhard, N. J. Planavsky, The rise of oxygen in Earth's early ocean and atmosphere. *Nature* **506**, 307–315 (2014).

74. N. L. Swanson-Hysell, The Precambrian paleogeography of Laurentia. *Ancient Supercontinents Paleogeogr. Earth* 109–153 (2021).
